# Comparative Analysis of Transcriptome and sRNAs Expression Patterns in the *Brachypodium distachyon*—*Magnaporthe oryzae* Pathosystems

**DOI:** 10.3390/ijms22020650

**Published:** 2021-01-11

**Authors:** Silvia Zanini, Ena Šečić, Tobias Busche, Matteo Galli, Ying Zheng, Jörn Kalinowski, Karl-Heinz Kogel

**Affiliations:** 1 Institute of Phytopathology, Centre for BioSystems, Land Use and Nutrition, Justus Liebig University (iFZ), 35392 Giessen, Germany; Silvia.F.Zanini@agrar.uni-giessen.de (S.Z.); Ena.Secic@agrar.uni-giessen.de (E.Š.); Matteo.Galli@agrar.uni-giessen.de (M.G.); Ying.Zheng@agrar.uni-giessen.de (Y.Z.); 2 Center for Biotechnology (CeBiTec), Bielefeld University, 33615 Bielefeld, Germany; tbusche@cebitec.uni-bielefeld.de (T.B.); joern@cebitec.uni-bielefeld.de (J.K.)

**Keywords:** argonaute, dicer, RNAi, plant disease, small RNA, virulence, gene expression

## Abstract

The hemibiotrophic fungus *Magnaporthe oryzae* (Mo) is the causative agent of rice blast and can infect aerial and root tissues of a variety of Poaceae, including the model *Brachypodium distachyon* (Bd). To gain insight in gene regulation processes occurring at early disease stages, we comparatively analyzed fungal and plant mRNA and sRNA expression in leaves and roots. A total of 310 Mo genes were detected consistently and differentially expressed in both leaves and roots. Contrary to Mo, only minor overlaps were observed in plant differentially expressed genes (DEGs), with 233 Bd-DEGs in infected leaves at 2 days post inoculation (DPI), compared to 4978 at 4 DPI, and 138 in infected roots. sRNA sequencing revealed a broad spectrum of Mo-sRNAs that accumulated in infected tissues, including candidates predicted to target Bd mRNAs. Conversely, we identified a subset of potential Bd-sRNAs directed against fungal cell wall components, virulence genes and transcription factors. We also show a requirement of operable RNAi genes from the DICER-like (DCL) and ARGONAUTE (AGO) families for fungal virulence. Overall, our work elucidates the extensive reprogramming of transcriptomes and sRNAs in both plant host (Bd) and fungal pathogen (Mo), further corroborating the critical role played by sRNA species in the establishment of the interaction and its outcome.

## 1. Introduction

The ascomycete fungus *Magnaporthe oryzae* (Mo) (anamorph: *Pyricularia grisea*) is the causal agent of rice blast, one of the most devastating and widespread diseases of cultivated rice, reducing yields up to 30% annually [1,2,3]. Members of the *Magnaporthe* genus can also infect a variety of other cereals, including barley, rye and wheat, making Mo a major threat to global food security [4,5]. Currently, blast management strategies rely on a combination of fungicide applications (e.g., azoles), development of resistant cultivars, and agronomic practices such as removal of crop residues, water management and crop/land rotation [6,7].

In the early stage of foliar infection Mo behaves as a biotroph, forming a biotrophic interfacial complex (BIC) between the primary invasive hyphae (derived from the penetration peg) and the infected host cell, where it secretes small molecules (effectors) to modulate the interaction [8,9]. Subsequently the fungus forms secondary hyphae and spreads to neighboring cells, undertaking a lifestyle change switching to a necrotrophic growth, with the appearance of the characteristic blast lesions on leaves [5]. Mo is able to infect all aerial parts of rice including nodes, panicles and necks, and has been shown to produce necrotic lesions on both rice and barley roots, although its lifestyle in roots seems to depend on the inoculation method [4,10]. Mo infections have also been established in *Brachypodium distachyon* (Bd), which has been proposed as a model for cereals as it is preferable for research over more complex crops such as wheat and barley [11,12]. Bd has a smaller genome (272 Mb in Bd21-3 v1.0 assembly) with low genome complexity, a short life cycle, simple growth requirements and a vast T-DNA insertion library available [13,14,15].

Small RNAs (sRNAs), such as small interfering RNAs (siRNAs) and micro RNA (miRNAs), are systemic signals that modulate distal gene regulation and epigenetic events in response to biotic and abiotic environmental cues in plants [16,17,18]. Particularly, sRNA-mediated gene silencing is one of the main defense mechanisms against viral attack and genome damaging effects of transposons. The action of sRNAs rests upon their role in RNA interference (RNAi), a conserved mechanism of gene regulation in eukaryotes at the transcriptional (TGS or transcriptional gene silencing) and post-transcriptional (PTGS or post-transcriptional gene silencing) levels [19,20,21]. In plants, the trigger for RNAi is either endogenous or exogenous (e.g., viral) double-stranded RNA (dsRNA) or hairpin RNA (hpRNA) that is processed into short 20 to 24 nucleotide (nt) double-stranded sRNAs by DICER-like (DCL) enzymes [22,23]. These sRNAs are incorporated into an RNA-induced silencing complex (RISC), containing an RNAse III-type endonucleolytic ARGONAUTE (AGO) protein to target complementary RNAs for degradation/inhibition or epigenetic modification by RNA-dependent DNA methylation (RdDM), histone modification and chromatin remodeling. Additionally, secondary sRNAs are generated in plants by RNA-dependent RNA polymerases (RdRPs) [21,24].

The genome of Mo encodes a complete RNAi machinery, comprised of two *DCL* genes, three *AGO* genes and three *RdRP* genes [25,26,27]. Knock-out (KO) of RNAi pathway components severely affected sRNA production in axenic culture, with deletion of *MoDCL2*, *MoRdRP2* and *MoAGO3* genes reducing sRNA expression levels. In particular, MoDCL2, but not MoDCL1, was necessary for siRNA production from dsRNA precursor molecules [27]. Of note, loss of MoAGO3 and MoRdRP1 function reduced both sRNAs and fungal virulence on barley leaves. sRNA-mediated alterations of TGS and PTGS have been detected *in vitro* both during starvation/different nutrient availability, and *in planta* during different stages of rice leaf infection [27]. Additionally, Mo sRNAs differentially accumulate in mycelia and appressoria [28], further supporting the notion that sRNAs regulate Mo’s development, growth and virulence. Similar to Mo, the formation of endogenous sRNAs in Bd is also subject to changes in response to external factors, where miRNAs have been proven to vary during exposure to abiotic stress and between vegetative vs. reproductive tissues [29], pointing to operable RNAi-based regulatory mechanisms in the model grass [30]. A total of 328 Bd miRNA precursor sequences have been identified so far and deposited in the miRBase database, corresponding to 536 mature miRNAs with predicted regulatory functions in cold [31], heat [32] and drought stress [33] and morphological alterations [34]. While knowledge about the RNAi machinery of Bd is not comprehensive and there is currently no data on RNAi KO mutants available from this organism, our recent work characterized the major RNAi genes *in silico*, resulting in 16 BdAGO-like and six BdDCL candidates [35]. Thus, the study showed that the RNAi machinery follows the trend that cereals have extended families of key enzymes involved in RNAi.

Consistent with the exchange of RNAs during animal-parasitic interactions [36,37,38], sRNAs are also exchanged in host plant–pathogen interactions. The plant–pathogenic fungus *Verticillium dahliae* (Vd) accumulated plant miRNAs when recovered from infected cotton samples, indicating that host-derived sRNAs were transmitted into the pathogen during infection [39]. Two of those cotton miRNAs, miR166 and miR159, target the fungal genes *Ca2+-dependent cysteine protease calpain (VdClp-1) and isotrichodermin C-15 hydroxylase (VdHiC-15),* respectively, which are known to contribute to fungal virulence. Similarly, *Arabidopsis* cells secrete vesicles to deliver sRNAs into grey mold fungal pathogen *Botrytis cinerea* (Bc), where they cause silencing of fungal genes critical for pathogenicity [40]. Consistent with the bidirectional move of sRNAs in plant-microbe interactions, Bc also produces sRNA effectors that downregulate *Arabidopsis* and tomato genes involved in immunity [41]. Some of these sRNAs, for example Bc-siR37, downregulate a large set of host immunity genes to enhance Bc pathogenicity [42].

Here we investigate the induction of sRNAs and transcriptome changes in the early stages of interaction of Bd and Mo based on data generated by parallel sequencing of sRNA and mRNA from infected leaf and root tissues. Using a previously published bioinformatics pipeline to characterize sRNA and its targets [43], we predict potential sRNA effectors whose targets were downregulated in the respective recipient organism.

## 2. Results

### 2.1. Requirement for Functional RNAi Genes in the Interaction of Brachypodium distachyon Bd21-3 and Magnaporthe oryzae 70-15

The *Magnaporthe oryzae* (Mo) 70-15 genome encodes three putative AGO and two DCL proteins, previously identified by domain search and phylogeny with known *Neurospora crassa* (Nc) RNAi machinery genes [25,26,27]. The corresponding protein sequences were obtained from NCBI: XP_003714515.1 (MGG_01541T0, MoDCL1), XP_003715365.1 (MGG_12357T0, MoDCL2), XP_003716704.1 (MGG_14873T0, MoAGO1), XP_003717504.1 (MGG_13617T0, MoAGO2) and XP_003714217.1 (MGG_01294T0, MoAGO3); they were included in new phylogenetic trees to corroborate previous findings (Figure S1A,B). To assess the conservation of key AGO and DCL domains, prediction was carried out with Simple Modular Architecture Research Tool (SMART). All three AGOs have conserved domain structures with five characteristic domains: N-terminal domain, DUF1785, PAZ (Piwi Argonaut and Zwille), L2 and PIWI (P-element Induced WImpy testis) (Figure S1C; Table S1). Additionally, multiple sequence alignment (MSA) confirmed the conservation of the DEDD catalytic tetrad (Asp/Glu/Asp/Asp) and the QF-V motif (Glu/Phe/Val) in the MoAGO PIWI domains (Figure S2). Likewise, both MoDCLs have the four conserved domains required for the cleavage of dsRNAs: DEXDc, HELICc, dicer-dimer and RIBOc (Figure S1D; Table S1). Subcellular localization of MoAGOs and MoDCLs was assessed using PSI-predictor; MoDCL1, MoAGO1 and MoAGO3 were predicted to primarily localize in the nucleus, while MoDCL2 and MoAGO2 were predicted to primarily localize in the cytosol. MoAGO1 and MoAGO2 also had secondary predicted localizations, the first in the cytosol and the second in plastids (Table S2).

Protein interaction analysis with STRING did not highlight any experimentally proven interaction for MoAGO and MoDCL proteins, but probable interactions could be inferred from data derived from homologs, found to either interact or co-express with several proteins in other species (Table S3A,B). In particular, all MoAGOs were predicted to interact with the two MoDCL proteins, a U5 small nuclear ribonucleoprotein component (MGG_13500) and a cell cycle control protein cwf14 (MGG_06309). For MoAGO1, additional interaction was predicted with Pumilio-family RNA-binding repeat protein (MGG_03158), ATP-dependent RNA helicase DED1 (MGG_02762), high-affinity glucose transporter (MGG_13651) and four uncharacterized proteins. In addition to interaction with AGO proteins, both fungal MoDCLs were predicted to interact with ATP-dependent DNA helicase MPH1 (MGG_04429), 30S ribosomal protein S16 (MGG_02598), WD domain-containing protein (MGG_06727) and three uncharacterized proteins.

**Figure 1:**
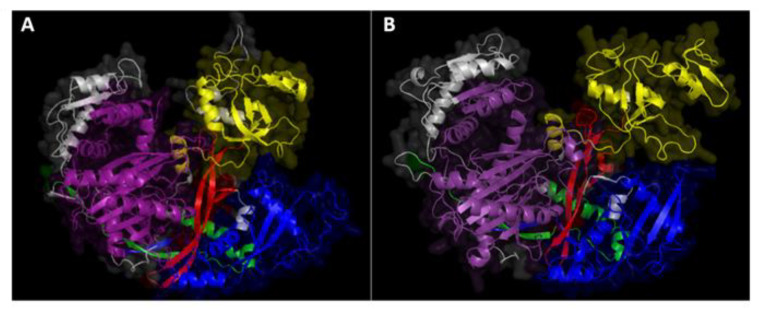
Predicted 3D structures of (**A**) *M. oryzae* MoAGO1 and (**B**) MoAGO3. Structures were modeled with SWISS-MODEL and domains were highlighted with PyMOL: blue = N-domain, red = DUF1785, yellow = PAZ (Piwi Argonaut and Zwille), green = L2, purple = PIWI (P-element Induced WImpy testis), the rest of the sequence with no domain predicted is colored in grey.

3D protein structure modeling for both MoDCL and MoAGO was performed with SWISS-MODEL. While no acceptable QMEAN Z-scores (>−4) values were obtained for the two DCLs and AGO2 models due to the lack of suitable reference structures, two models for AGO1 (model 1 based on hAGO1, GMQE = 0.52 QMEAN = −3.98 and model 3, based on hAGO2, GMQE = 0.51 QMEAN = −3.31) and model 6 for AGO3 (based on hAGO4 template, GMQE = 0.46 QMEAN = −3.50) passed the first quality check and were further validated with PROCHECK and WHATCHECK. Model 1 of AGO1 had a higher percentage of residues in the core region of the Ramachandran plot (88.4%) and a better Ramachandran Z-score (−1.808) compared to model 3 (87.6% and −1.531 Z-score), while the AGO3 model scored 86.7% and −2.088, respectively. As a result, model 1 of AGO1 and model 6 of AGO3 were selected as the best models and visualized with PyMOL (Figure 1A,B).

**Figure 2:**
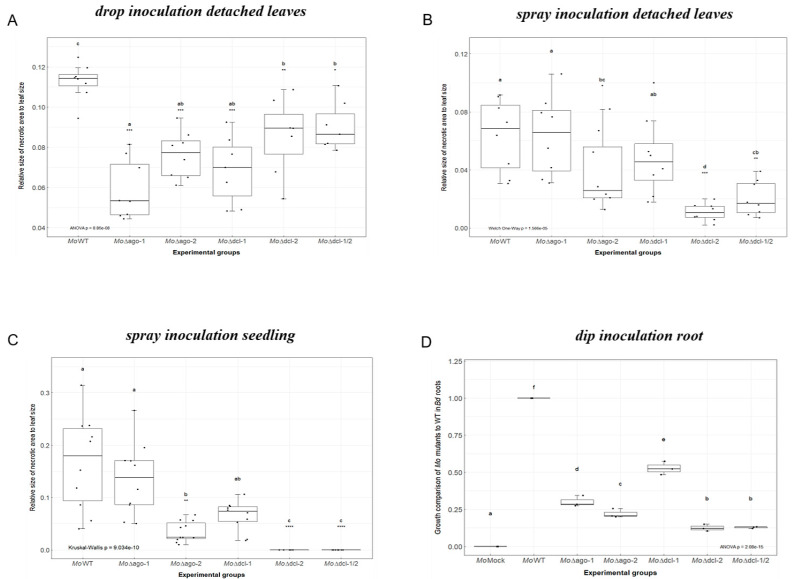
Infection assays of *M. oryzae* RNAi mutants. (**A**) Detached second youngest leaves of three-week-old Bd seedlings were drop inoculated with 10 μL Mo conidia suspension 50 × 10^3^ spores mL^−1^ in 0.002% Tween20 and kept under high humidity at 16 h light/8 h dark cycle at 22 °C/18 °C. Fungal pathogenicity was assayed via ImageJ software at 5 DPI. The experiment was conducted two times (*n* = 8 plants per experimental group) with similar results. Comparisons between groups was performed via ANOVA and Tukey’s range test for multiple comparisons. (**B**) Detached Bd leaves were spray inoculated with a total of 250 μL Mo conidia suspension 50 × 10^3^ spores mL^−1^ in 0.002% Tween20 and kept and evaluated as in (A). The experiment was conducted two times (*n* = 8 plants per experimental group) with similar results. Comparisons between groups was performed via Welch one-way test coupled with pairwise t-tests with Benjamini–Hochberg *p*-value adjustment. (**C**) Three-week-old Bd seedlings were spray inoculated with Mo conidia suspension 120 × 10^3^ spores mL^−1^ in 0.002% Tween20 and kept and evaluated as in (A). The experiment was conducted two times (*n* = 18 plants per experimental group) with similar results. Comparisons between groups was performed via Kruskal–Wallis test and Dunn’s test of multiple comparisons. (**D**) Roots of seven-day-old Bd seedlings were inoculated with 1 mL of Mo conidia suspension 125 × 10^3^ spores mL^−1^ in 0.002% Tween20 for 3 h. Afterwards, seedlings were transplanted in small pots (3 cm Ø) and grown for an additional 5 days before harvesting. Fungal amount was calculated by qPCR based on the ratio of fungal actin (*MoActin*). The experiment was conducted two times (*n* = 6 roots per experimental group) with similar results. Comparisons between groups was performed via ANOVA and Tukey’s range test for multiple comparisons. (**A**–**D**) Letters represent statistical difference among all groups’ means (α < 0.05). Asterisks represent statistical difference of each group against wildtype (wt) (* *p* < 0.05; ** *p* < 0.01; *** *p* < 0.001; **** *p* < 0.0001).

Next, we assessed the virulence of Δ*moago1,* Δ*moago2*, Δ*modcl1*, Δ*modcl2* and the double mutant Δ*modcl1/2* using Bd21-3 as a host. Negative effects on vitality of the five mutants vs. Mo wildtype (wt) could not be detected when inspecting conidia germination and appressorium formation on poly-L-lysine coated glass slides by 24 h after the onset of germination (Figure S3). In contrast, virulence of these mutants was compromised both on Bd leaves and roots (Figure S4). Since we expected differences in infection phenotypes depending on test conditions, three different leaf inoculation protocols were tested: (i.) upon drop inoculation of detached leaves all mutants showed significant (Kruskal–Wallis test, *p* < 0.05) reduction in necrotic lesion size, ranging from a −20% to −48% reduction of relative necrotic area compared to Mo wt (Δ*moago1* −48%; Δ*moago2* −32%; Δ*modcl1* −39%; Δ*modcl2* −24%; Δ*modcl1/2* −20%; Figure 2A); (ii.) upon spray inoculation of detached leaves, virulence of Δ*moago1* and Δ*modcl1* was virtually unaffected as they showed the typical necrotic lesions at 5 days post inoculation (DPI), compared to Δ*moago2*, Δ*modcl2* and Δ*modcl1/2*, showing significantly reduced necrotic lesion areas (compared to Mo wt: Δ*moago2* −40%; Δ*modcl2* −83%; Δ*modcl1/2* −68% necrotic lesion sizes; Figure 2B); (iii.) similar phenotypes were observed in the whole seedling spray assay, with the exception of Δ*modcl2* and Δ*modcl1/2* that appeared avirulent and did not show any detectable lesions (reduction in the necrotic lesion size compared to Mo wt: Δ*moago2* −79%; Δ*modcl2* −100%; Δ*modcl1/2* −100%; Figure 2C). Finally, mutants were inoculated on Bd roots and fungal presence was measured at 5 DPI by qPCR based on the fungal actin. All mutants showed a significant reduction in fungal biomass, with Δ*moago2*, Δ*modcl2* and Δ*modcl1/2* being the most severely affected (reduction in *MoActin* levels compared to wt: Δ*moago1* −70%; Δ*moago2* −78%; Δ*modcl1* −47%; Δ*modcl2* −87%; Δ*modcl1/2* −87%; Figure 2D). These results confirm and extend previous findings that Mo’s RNAi machinery is required for disease development in cereals and, in particular, that DCL2 is a key factor not only for sRNA biogenesis but also for fungal virulence.

### 2.2. Differentially Expressed Genes (DEGs) in the Early Stages of Leaf and Root Infections

**Figure 3:**
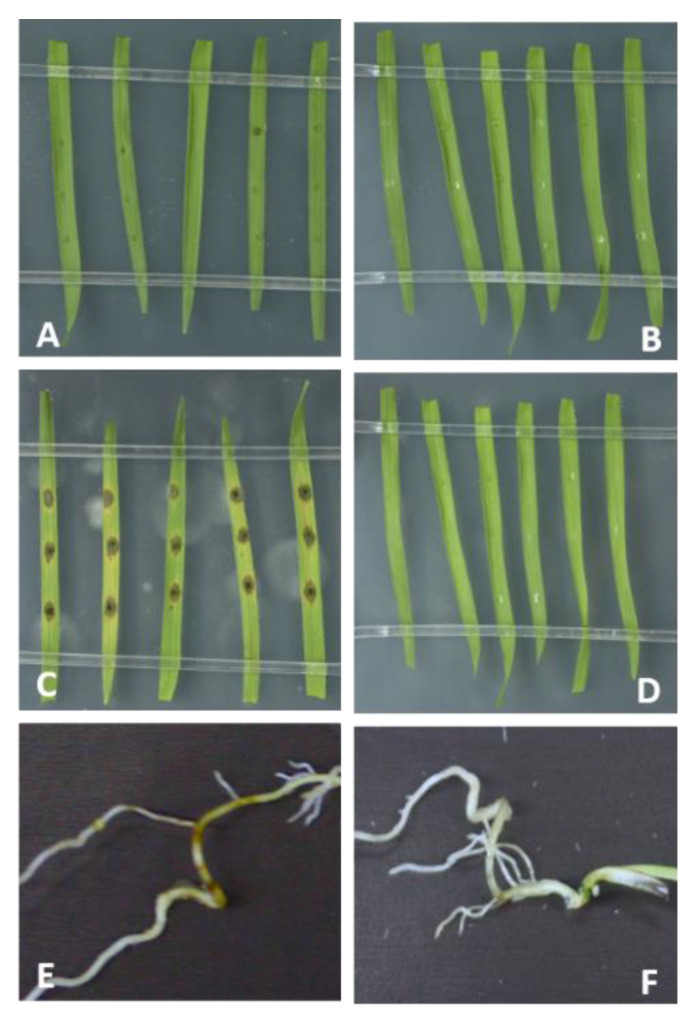
Brachypodium blast caused by *M. oryzae* Mo 70-15 on leaves and roots of *B. distachyon* Bd21-3. (**A**,**D**) Detached 21-day-old Bd leaves were drop-inoculated with 10 μL of Mo suspension (50,000 conidia/mL) and kept for (**A**) 2 and (**C**) 4 days at high humidity, or mock-inoculated (**B**,**D**) with Tween water. Roots of 7-day-old Bd seedlings were (**E**) drop-inoculated with 1 mL of Mo suspension (250,000 conidia/mL) or (**F**) mock-inoculated or and kept for 4 days under high humidity at 16 h light/8 h dark cycle at 22 °C/18 °C.

**Figure 4:**
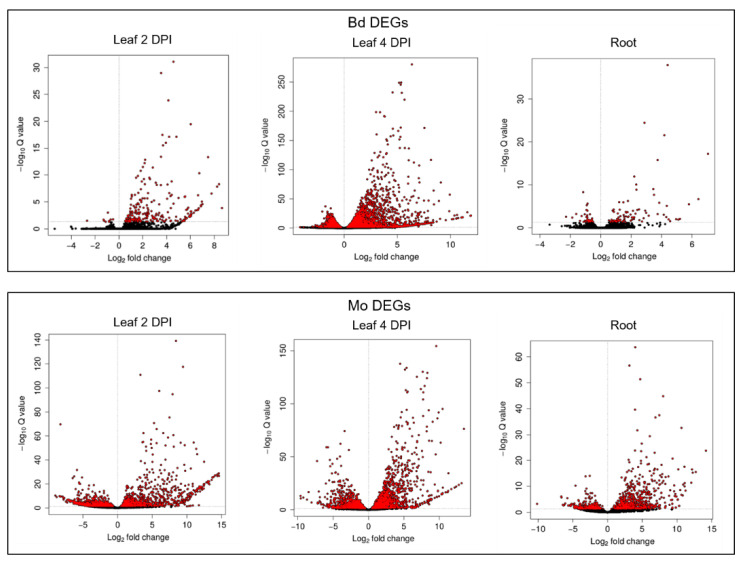
Volcano plots of DESeq2 results based on mRNAseq analysis of *M. oryzae*-infected leaves and roots of *B. distachyon* vs. control. Differentially expressed genes (DEGs) are highlighted in red with significant adjusted *p*-values (padj < 0.05).

TruSeq^®^ Stranded mRNA libraries were produced from (i.) Mo axenic culture, (ii.) Mo-infected and mock-treated Bd roots (4 DPI) and (iii.) Mo-infected and mock-treated Bd leaves (2 DPI and 4 DPI) (Figure 3). These early time points were chosen to assess gene expression patterns both in the foliar biotrophic and necrotrophic growth phase of hemibiotrophic Mo [5]. mRNAseq analysis revealed a total of 233 Bd-DEGs (Wald test, Benjamini–Hochberg (BH) adjustment, adjusted *p*-value (padj) < 0.05) in leaves at 2 DPI (224 upregulated and 9 downregulated), compared to 4978 at 4 DPI (3023 upregulated and 1955 downregulated) and 128 in roots (89 upregulated and 39 downregulated) (Figure 4; Table 1; Figure S5).

**Figure 5:**
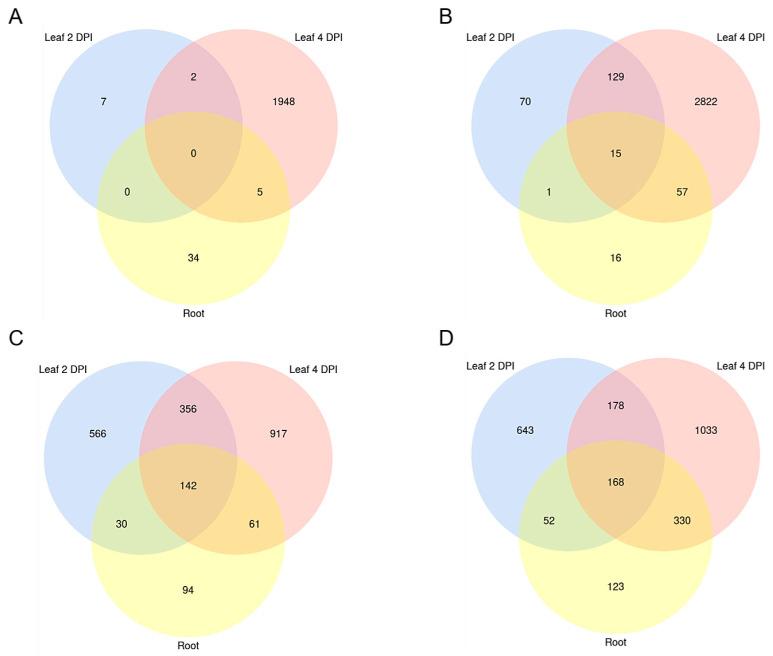
Venn diagram of differentially expressed *B. distachyon* and *M. oryzae* genes. Significantly (**A**) downregulated (fold change (FC) < 0 padj < 0.05) and (**B**) upregulated (FC > 0 padj < 0.05) Bd genes shared between setups. Significantly (**C**) downregulated (FC < 0 padj < 0.05) and (**D**) upregulated (FC > 0 padj < 0.05) Mo genes shared between setups (“Leaf 2 DPI”, “Leaf 4 DPI” and “Root”). Transcript downregulation was calculated from mRNAseq data with DESeq2.

Minor overlaps were observed comparing downregulated Bd genes between the biological setups, with only two shared between the foliar infections, and five between the leaf (4 DPI) and the root setup (Figure 5A). Among the upregulated genes, the highest overlap was seen between the foliar infections, sharing 134 DEGs, compared to 16 DEGs shared between root and leaf 2 DPI, and 72 between root and leaf 4 DPI (Figure 5B). Interestingly, 15 Bd genes were upregulated in all setups, including defense-related *ABC transporter* (BdiBd21-3.3G0465100.1), *anthranilate synthase component II* (BdiBd21-3.5G0159100.1), *protein kinase xa21* (BdiBd21-3.3G0144800.1) and *secologanin synthase-like* (BdiBd21-3.2G0563800.1) (Table 2).

In total, we found 2135 Mo DEGs (Wald test, Benjamini–Hochberg adjustment, padj < 0.05) in leaves at 2 DPI (1041 up and 1094 down), 3186 DEGs at 4 DPI (1710 up and 1476 down) and 1000 DEGs in infected roots (673 up and 327 down) (Figure 4; Table 1; Figure S5). A notable overlap of fungal DEGs was detected between the two foliar infections, with 346 up- and 498 downregulated genes shared, compared to 220 up- and 172 downregulated DEGs shared between leaf 2 DPI and root infection, and 498 up- and 203 downregulated DEGs shared between leaf 4 DPI and root infection. Overall, 142 genes were significantly downregulated in all setups, while 168 were consistently upregulated in all samples, including genes involved in fungal development, metabolism and pathogenicity (Figure 5C,D).

**Figure 6:**
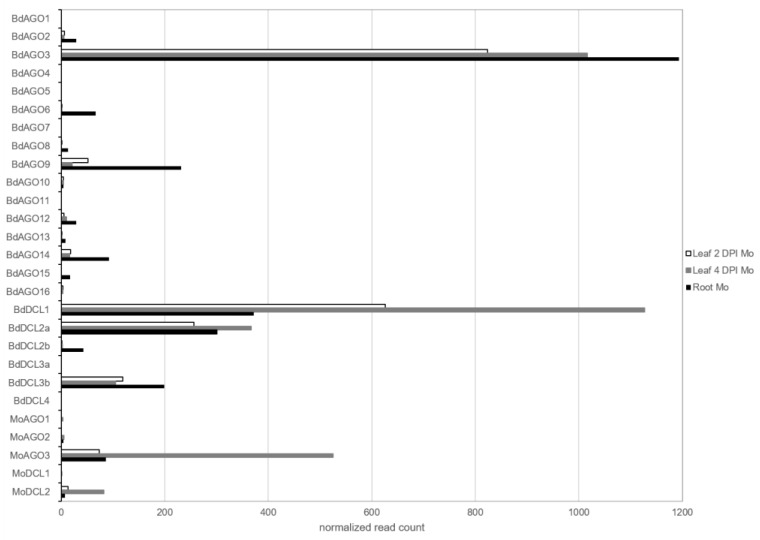
Expression of predicted *ARGONAUTE (AGO)* and *DICER-like (DCL)* during the interaction of *B. distachyon* and *M. oryzae* from mRNAseq results. Normalized read counts of each RNAi component were retrieved from DESeq2 analyses of infected datasets: leaf 2 DPI (white), leaf 4 DPI (grey) and root (black).

Expression of *DCL* and *AGO* genes was assessed in both organisms in leaves and roots, which showed that *BdDCL1* and *MoDCL2* had the highest expression among the DCL family, and *BdAGO3* and *MoAGO3* among the AGO family (Figure 6).

Interestingly, *BdAGO9*, which is the closest homolog to *AtAGO1* [35], was detected expressed in all three setups and had the highest read count of the AtAGO1-like clade. Moreover, *BdAGO1*, *BdAGO5* and *BdAGO15* were only expressed in the root samples, while *BdAGO4*, *BdAGO7*, *BdAGO11*, *BdDCL3a* and *BdDCL4* were not detected in any of the datasets. Finally, the majority of *DCL* and *AGO* genes were not differentially regulated during the infection, with the exception of *BdDCL3b* and *BdAGO3* (minor downregulation; >−1 log_2_FC) and *BdDCL1-2a* and *MoAGO3* (minor upregulation; <1 log_2_FC) (Table S4).

### 2.3. Gene Ontology Enrichment (GOE) and Defense-Related Gene Expression in Mo-Infected Bd

GOE analysis using AgriGO v2 was performed on all Bd DEGs identified in leaves and roots by mRNAseq. In all three datasets, GO:0003824 (catalytic activity), GO:0016491 (oxidoreductase activity) and GO:0044710 (single-organism metabolic process) were enriched (Table S5). The highest number of Gene Ontology (GO) terms enrichment—in particular those related to various cellular metabolic processes—was recorded in leaves at 4 DPI, which corresponds to the necrotrophic fungal growth phase (Figure S6). Consistent with the extensive metabolic reprogramming highlighted in the GOE analysis, the highest number of DEGs also was observed in these samples. Specifically, we detected significant upregulation of known Bd defense response genes such as *pathogenesis-related 1 (PR1), PR3 and PR5*, receptor-like protein kinases and *Myb* and *WRKY* transcription factors (Table 2). Only minor overlap was observed between defense-related genes expression patterns in the leaf 2 DPI and root samples. Three predicted PR proteins were upregulated in both 4 DPI and root setups, including PR10 and PRB1-2 (log_2_FC > 3 in both, while not induced at 2 DPI) and two were found significantly upregulated in the two foliar datasets, including PR1 (with a log_2_FC > 6 in these datasets and not induced in the roots).

### 2.4. GOE and Gene Expression in Mo during Bd Infection

Next, significantly up- and downregulated Mo genes were subjected to GOE analysis, showing the highest number of enriched GO terms in the root samples, including several terms relating to the interaction with the host (Table S6; Figure S7). Overall, many infection-related genes were significantly upregulated, including effectors *AVR Pita1* and *PWL2 (Pathogenicity toward Weeping Lovegrass)* and *NOXs (superoxide-generating NADPH oxidases)* (Table 3).

### 2.5. sRNA Reprogramming in Bd and Mo at Early Infection Stages

**Figure 7:**
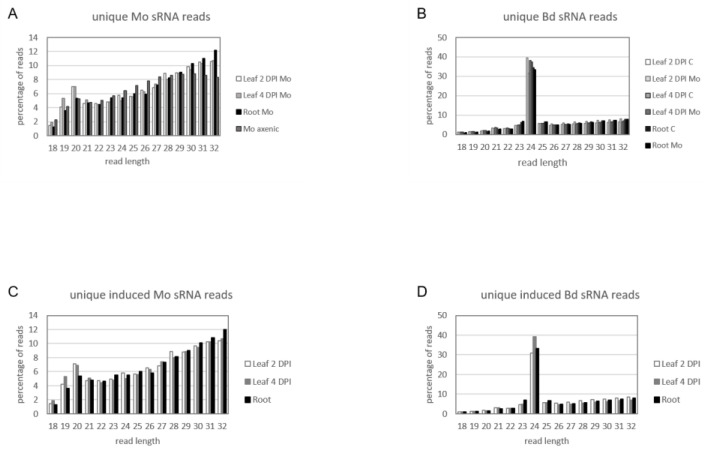
Size distribution of unique sRNA reads in the interaction of *M. oryzae* and *B. distachyon*. Relative size distribution (in percentage) of unique filtered sRNA reads assigned to (**A**) Mo or (**B**) Bd. Reads were assigned to either Mo or Bd only if aligning 100% to the organism of origin genome and had at least two mismatches to the interacting organism genome. (**C**,**D**) Relative size distribution of unique filtered sRNA reads assigned to (**C**) Mo or (**D**) Bd and induced or increased in infected samples compared to controls (i.e., axenic fungal cultures and non-inoculated plants, respectively). Samples for sRNA sequencing by Illumina HiSeq1500 were taken from different setups: leaf biotrophic phase (“2 DPI”), leaf necrotrophic phase (“4 DPI”) and root (“root”).

**Figure 8:**
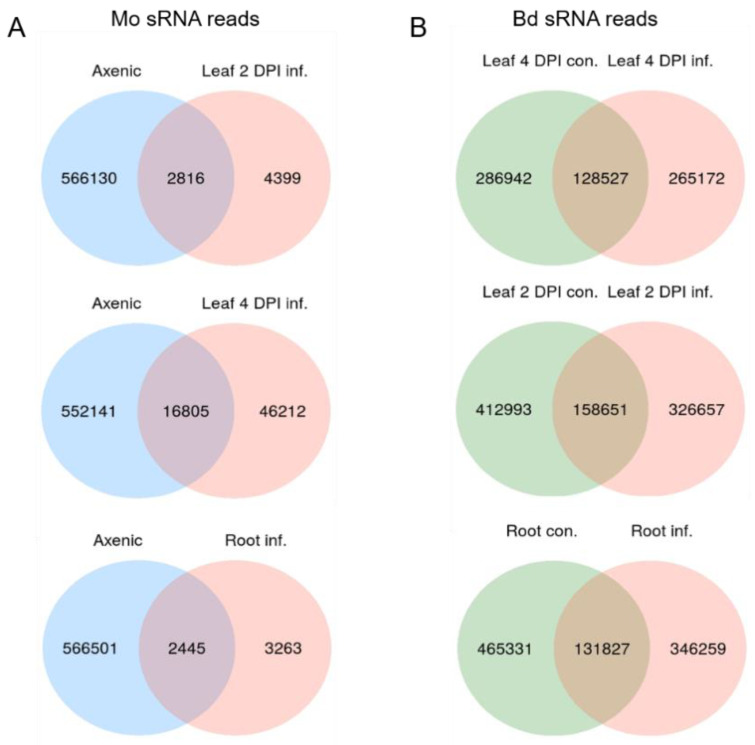
Venn diagrams of unique filtered sRNAs reads from *M. oryzae* and *B. distachyon*. (**A**) Venn diagram of Mo sRNA reads (18–32 nt) from axenic culture and Mo-infected Bd leaves (2 DPI, 4 DPI) and roots (4 DPI). (**B**) Venn diagram of Bd sRNA reads (18–32 nt) in non-infected and Mo-infected Bd leaves.

We assessed the expression of sRNAs in the same biological material utilized for mRNAseq. Before high-throughput next generation sequencing (NGS), multiplexed sRNA libraries were size selected for 15 to 35 nt fragments (140–160 nt including TruSeq adapters). Single end sequencing on Illumina HiSeq1500 platform generated between 22 million (mil) and 38 mil reads each (Table S7). Quality check of raw reads was performed with FastQC, adapters were removed with cutadapt and reads were size selected between 18 to 32 nt. The organism of origin of the trimmed reads was predicted by mapping via Bowtie alignments to both Bd21-3 and Mo 70-15 genomes [44] (Bd21-3 v1.1 DOE-JGI, http://phytozome.jgi.doe.gov/). Ambiguous reads that could not be assigned to the organism of origin with high confidence were excluded to avoid miscalling. As expected, most reads in Mo-infected samples were assigned to Bd (with 100% match) and not to the fungus (with at least two nucleotide mismatches) (Table S7). Size distribution of genome matched unique sRNA reads followed a similar trend throughout samples, with the Mo reads showing a minor peak around 20 nt and Bd reads a considerable peak at 24 nt (Figure 7A,B). Fungal unique sRNA reads were then compared among datasets derived from Mo axenic culture and Mo-infected plant tissues and classified as either exclusive or shared between samples (Figure 8A). Some 5708 Mo-sRNAs were identified in Mo-infected roots, of which 3263 (57.15%) were found exclusively in the infected sample and not in the axenic culture. A total of 7215 Mo-sRNAs were identified in infected leaves during the biotrophic phase (2 DPI), of which 4399 (60.97%) were found exclusively in infected samples. Finally, a total of 63,017 were found in infected leaf tissue during the necrotrophic phase (4 DPI), of which 46,212 (73.33%) were exclusively found in infected samples.

Next, we compared unique Bd-sRNA reads in leaf and root samples from Mo-infected and mock-treated Bd (Figure 8B). A large number of sRNAs were found in infected tissues: 571,644 in leaves at 2 DPI, of which 326,657 (72.24%) were specific for infection; 415,469 at 4 DPI, of which 265,172 (69.06%) were specific for infection; and 597,158 Bd-sRNAs in infected roots, of which 346,259 (77.92%) were specific for infection. These data show that the majority of unique sRNAs of both interacting organisms are expressed exclusively during the interaction, suggesting that they are relevant to the establishment and outcome of the disease. We further selected unique Bd-sRNAs that (i.) were either found exclusively in infected plant tissues or (ii.) showed higher reads in infected vs. mock-inoculated tissue. Interestingly, the size distribution of these induced Bd-sRNA did not show a change in length preference compared to the total unique reads (Figure 7C,D).

### 2.6. Infection-Related Bd miRNAs

Filtered Bd-sRNA reads were analyzed with Shortstack 3.8.5 to identify infection-related upregulation of potential plant miRNAs. The program identified 14 putative miRNA-generating loci in leaves at 2 DPI, 15 at 4 DPI and 16 in the roots. Matching predicted miRNA generating clusters in the uninfected samples were identified via their genomic coordinates, and numbers of total reads per cluster were compared between infected and uninfected samples to select exclusively expressed or upregulated miRNA loci. The selected clusters’ sequences were aligned to known Bd miRNA stem loop precursor sequences obtained from the miRBase database: three known miRNAs were upregulated in leaves at 2 DPI, six at 4 DPI and four in the roots (Table 4). Structures of the miRNA precursors are shown in Figure S8.

### 2.7. In Silico Prediction of Mo-sRNA Targeting Bd Transcripts

**Figure 9:**
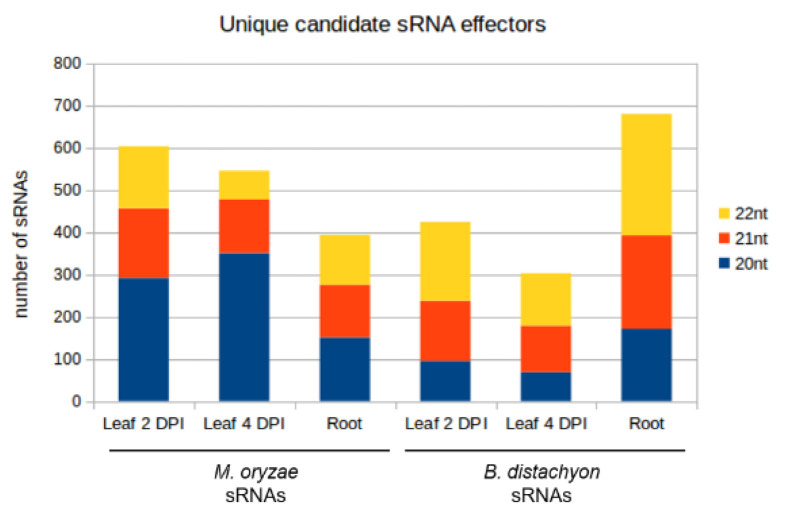
Size composition of unique candidate sRNA effectors from *M. oryzae* and *B. distachyon*. The number of unique reads is reported on the y-axis.

**Figure 10:**
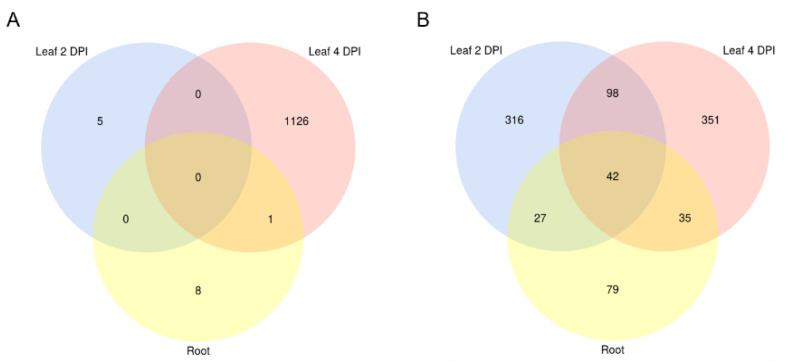
Venn diagram of downregulated predicted RNAi targets in *M. oryzae* and *B. distachyon*. Significantly downregulated (FC < 0 padj < 0.05) (**A**) Bd mRNA targets with complementarity to putative Mo sRNAs effectors shared between setups. (**B**) Mo mRNA targets with complementarity to putative Bd sRNA effectors. Setups: Leaf biotrophic phase (2 DPI) Leaf necrotrophic phase (4 DPI), and root. Transcript downregulation was assessed from mRNAseq data with DESeq2.

sRNAs that were either exclusively produced or increased in infected tissues and had a normalized read count of at least five reads per million (RPM) were further investigated. Induced 20–22 nt sRNAs originating from non-coding regions of the Mo 70-15 genome (Figure 9) were selected as candidate cross-kingdom effectors with potential targets in the Bd transcriptome. Target prediction for these Mo-sRNAs was performed with psRNATarget using modified settings and a default score cut-off of 5.0. Target prediction data were then compared with the DEGs information derived from the concomitant mRNA sequencing. Of the total downregulated Bd transcripts, five were predicted to be targeted by Mo-sRNAs in the leaf 2 DPI setup, 1128 in the 4 DPI setup and 10 in the root (Table 5), with only one target shared between the root and the leaf 4 DPI datasets (Figure 10A). Predicted plant targets in leaves (4 DPI) included transcription factors such as *transcriptional regulator algH*, *myb-related protein Zm38* and *NIGTH1*, along with aquaporin transporters and RNA helicases, including the putative *BdDCL3b* (BdiBd21-3.2G0305700), along with resistance genes *RGA3* (BdiBd21-3.2G0771500.1), *RGA4* (BdiBd21-3.3G0396200.1) and *RPP13-like protein 3* (BdiBd21-3.3G0776700.1) (Table 6).

### 2.8. In Silico Prediction of Bd-sRNAs Targeting Mo Transcripts

Given that plant-derived sRNAs can move into fungal pathogens during fungal infections [39,40], we further explored the possibility that Bd-sRNAs have corresponding downregulated Mo transcripts. In total, 20–22 nt Bd-sRNAs (Figure 9) that (i.) originate from the non-coding regions of the Bd21-3 genome, and (ii.) show a higher read count in the Mo-infected compared to non-infected sample were selected. We found 424, 302 and 681 Bd-sRNA in leaves at 2 DPI and 4 DPI and in roots, respectively, with predicted targets in the Mo transcriptome (Table 5). In the next step, we selected for targets found downregulated in the mRNAseq analysis, which further reduced the predicted Mo ck-sRNAs to 314 in leaves at 2 DPI, 236 at 4 DPI and 263 in roots, respectively, corresponding to potentially targeted mRNAs downregulated in leaves at 2 DPI (484), 4 DPI (527) and roots (183) (Table 5). Downregulated Mo targets include cell wall-related genes such as *chitin deacetylase 1* (MGG_05023), *cell wall protein* MGG_09460 and virulence genes, such as *CAP20* (MGG_11916) (Table 7). By comparing predicted Mo targets in infected tissues, we found a considerable overlap between leaf and root samples (42 Mo mRNAs) and between the two leaf samples (140 Mo mRNAs) (Table 7; Figure 10B).

We searched the Pathogen-Host Interactions Database (PHI-base) for available information on loss-of-virulence mutants for the predicted Mo target genes. A list of downregulated predicted Mo targets and corresponding PHI-base phenotypes is shown in Table 8. Of note, the predicted Mo genes are involved in virulence and pathogenicity, including *CON7* transcription factor (MGG_05287) predicted to be targeted in the Mo-infected root, the effector *AvrPiz-t* (MGG_09055) predicted to be targeted in the leaf at 4 DPI and the *vacuolar glucoamylase SGA1* (MGG_01096) predicted to be targeted in the leaf at 2 DPI. Additionally, potential targets shared between more than one setup included (MGG_00620), *Sso1* (MGG_04090) encoding a SNARE protein and *YHM2* (MGG_07201) encoding mitochondrial DNA replication protein (2 DPI leaf vs. root), as well as *MoATG17* (MGG_07667) encoding an autophagy-related protein (biotrophic vs. necrotrophic leaves), whose respective mutants are also known to be compromised in pathogenicity [45,46] (Table 7 and Table 8).

## 3. Discussion

Based on the work done to elucidate the function and 3D structure of *Brachypodium distachyon* AGO and DCL proteins published by Šečić et al. [35], we set out to investigate these RNAi machinery components in *Magnaporthe oryzae*. Mo has being extensively studied as a model for RNAi in fungi, and it is known to encode three AGOs, two DCLs and three RdRPs [25,26]. Interestingly, while the phylogeny with other fungal RNAi machineries such as *N. crassa, Mucor circinelloides, Cryphonectria parasitica* and *Schizosaccharomyces pombe* and the domain conservation have been reported, we found no information regarding the proteins’ predicted interactome, subcellular localization and 3D protein structures, which would help elucidate the function and relevance of these proteins in vivo. We confirmed that all three fungal AGOs have the characteristic PAZ (Piwi Argonaut and Zwille) and PIWI (P-element Induced WImpy testis) domains: the first is involved in the recognition of the 3′ end of the guide sRNA molecule [47], while the second is an RNase H domain that confers AGOs the ability to cleave single-stranded RNAs complementary to the guide sRNA sequence [48]. A closer look at the PIWI domains via MSA confirmed the conservation of both the QF-V motif, involved in the sorting of sRNAs based on their sequence and secondary structure [49] and the DEDD catalytic tetrad, critical for the protein ‘slicer’ activity [48]. Similar to NcDCLs, both MoDCLs lacked predicted PAZ domain but differed from the *N. crassa* ortholog showing predicted dicer-dimer domains and lacking predicted dsRNA-specific ribonuclease and ds-RNA binding (DSRM) domains [27]. At this point of the analysis, no major differences were recorded among either Mo protein families, which would help elucidate the differences in both sRNA production and virulence reported in the RNAi KO mutants by Raman et al. [50]. Interestingly, protein subcellular localization helped shed additional light on MoAGO3 and MoDCL2 non-redundant roles within their families, with MoAGO3 predicted to localize exclusively in the nucleus, compared to MoAGO1, predicted in both the nucleus and the cytosol, and MoAGO2, predicted to localize in the cytosol and plastid. Moreover, we predicted MoDCL2 to exclusively localize in the cytosol, while MoDCL1 in the nucleus, confirming the distinct roles for these proteins in contrast with their redundant function *N. crassa* [51]. Additionally, predicted interactomes of MoAGOs showed significant differences between MoAGO1 vs. MoAGO2 and MoAGO3, which would point to a unique role of this protein. Specifically, the predicted interaction with an ATP-dependent RNA helicase DED1 (MGG_02762) and a Pumilio-family RNA-binding repeat protein (MGG_03158) would suggest a unique role for MoAGO1 in translation regulation [52,53]. KO mutants of these RNAi components have been previously characterized both for their sRNA accumulation patterns and their virulence on barley [27,50]. Except for Δ*moago3*, which did not sporulate and could not be further utilized, we tested the morphology and virulence of the available mutants in our newly established Bd pathosystems. While all five RNAi mutants showed unaltered growth, morphology and conidiation in axenic cultures, we detected significant differences in their ability to infect Bd tissues. Interaction between plants and its host pathogen is a dynamic system, influenced by several genetic and environmental factors which may alter the course of the infection and its reproducibility. The inoculation method proved to be critical for the assessment of virulence alterations, with the drop inoculation on detached leaves resulting in the most stable assay, and the spray on whole seedlings resulting in the most extreme phenotypes, with Δ*modcl2* and Δ*modcl1/2* showing a complete loss of pathogenicity. These results both confirm and expand the key role of MoDCL2 first hypothesized by Raman and colleagues in a variety of fungal biological processes, including: sRNA biogenesis, fungal development and, as shown here, fungal virulence [27,50]. The reduction of virulence observed among RNAi mutants throughout the different setups further substantiate the importance of a fully functioning fungal silencing machinery and sRNAs generated for a successful infection of Bd tissues.

GOE analysis of Mo DEGs highlighted an enrichment of terms mainly related to fungal growth and development in all infected tissues. Especially in the root GO, terms related to interactions with other organisms, particularly via protein secretion, were found overrepresented. By assessing expression of protein effectors, we confirmed upregulation of known avirulence genes, including *AVR Pita1* (coding for a metalloprotease) and *PWL2* (a glycine-rich protein recognized by weeping lovegrass and finger millet R proteins), both found highly upregulated in the foliar biotrophic phase (2 DPI), and *Avr-Pik/km/kp*, upregulated at 2 and 4 DPI.

Genes involved in appressorium and penetration peg functionality were upregulated in all three setups, including *WISH* (Water wettability, Infection, Surface sensing and Hyper-conidiation) G-protein coupled receptor protein, whose KO renders the fungus unable to develop appressoria and establishes the infection on intact rice leaves [54] and superoxide-generating NADPH oxidases *NOX1* (MGG_00750) and *NoxD* (MGG_09956). NOX1 is involved in cell wall biogenesis, affecting both chitin and melanin biosynthesis and deposition. KO of this gene resulted in a complete loss of appressorium-mediated cuticle penetration and failure of *in planta* proliferation even when inoculated onto wounded rice seedlings [55]. Similar defects were detected in rice leaves and roots inoculated with Δ*noxD* [56], confirming the key role played by NOX proteins not only in growth and sexual reproduction, but also in fungal virulence. *PLC3*, significantly upregulated at both timepoints in leaves, is also required for normal conidiation and appressorium function, but contrary to NOXs normal infection was observed when Δ*moplc3* was inoculated by infiltration into wounded rice leaves, excluding a function of PLC3 in fungal growth once inside host cells [57]. As expected, we detected in the later timepoint of the leaf infection (4 DPI) upregulation of genes known to be required for infection maintenance and expansion. One of these genes, *S-(hydroxymethyl) glutathione dehydrogenase* (MGG_06011), was exclusively upregulated in the 4 DPI dataset and it is known to be involved in the growth of infectious hyphae on barley leaves [58].

Overall, our analysis shows that Mo undergoes an extensive reprogramming during the establishment and maintenance of the infection and highlights the commonalities and differences in expression patterns depending on the inoculated tissue and the progression of the colonization. Of note and consistent with the detection of necrotrophic lesions on Bd roots (Figure S4), fungal reprogramming during the root infection is closer to the one reported in the foliar necrotrophic phase (with 701 DEGs shared between the setups) compared to the biotrophic phase (only 392 DEGs shared between the setups).

mRNA sequencing of Mo-infected and non-infected Bd leaves and root samples allowed for a systemic analysis and comparison of expression pattern alterations of Bd genes in response to the fungal pathogen. As predicted, the highest amount of DEGs was observed at 4 DPI in leaves, when the infection is spreading outside of the inoculation point and the fungus has switched to a more aggressive necrotrophic lifestyle [5,12,59]. The relatively low amount of DEGs in the other two setups is consistent with the early infection stages and the limited amount of fungal biomass both in the root and at 2 DPI in leaves. Interestingly, in all three setups the percentage of upregulated DEGs was higher compared to downregulated DEGS, with 96% upregulated in leaves at 2 DPI, 60.7% at 4 DPI and 69.5% in the roots, indicating an overall strong induction of gene expression. Consistent with the highest numbers of DEGs detected at 4 DPI, most GO terms reported in Table S5 belong to this dataset, with terms related to metabolic and biosynthetic processes being the most prevalent. Interestingly, all datasets had enriched terms related to oxidoreductase activities (GO:001649) confirmed also by the upregulation of BdiBd21-3.4G0171000, coding for a multicopper oxidase, in all setups, and BdiBd21-3.1G0233800 and BdiBd21-3.1G0233900, coding for peroxidases, in the leaf 4 DPI datasets. Peroxidases are commonly associated with plant responses to stress and specifically fungal infection, as they are involved in a variety of processes including the synthesis of phenols such as tannins and melanins, reactive oxygen species (ROS) removal, lignin biosynthesis and induction of defense responses by stimulating intracellular Ca2+ signaling [60]. Another gene upregulated in all three setups is *secologanin synthase-like* (BdiBd21-3.2G0563800), encoding an enzyme involved in the biosynthesis of monoterpenoid indole alkaloids (MIAs), also reported upregulated in Bd following *F. pseudograminearum* infection [61]. Expression of other defense-related genes was induced in one or more of our datasets, including known pathogenesis-related proteins, receptor kinases, transcription factors and ABC transporters. Once again, 2 DPI and root samples were found overlapping in only a few of these genes. Examples of genes consistently found upregulated in all three setups are BdiBd21-3.3G0144800, encoding a protein kinase xa21 which confers resistance to *Xanthomonas oryzae pv. oryzae* race 6 [62], an *ABC transporter A family member 2-like* (BdiBd21-3.3G0465100) and the *pleiotropic drug resistance protein 3-like* (BdiBd21-3.2G0550500).

Interestingly, all *PR* genes shown in Table 5 were upregulated in leaves at 4 DPI, while leaf 2 DPI and root PR protein expression patterns did not overlap, with only two (PR-like and PR1-like) upregulated at 2 DPI, and three (two PR1-like and PR10) in the root datasets. Similarly, the strongest and most widespread upregulation in the other pathogen sensing/defense-related genes was observed at 4 DPI, with an upregulation of transcription factors belonging to MYB, WRKY and NAC families consistent with the upregulation observed in Bd after *F. pseudograminearum* infection [61], and the knowledge that these families regulate a variety of plant responses, including those to biotic stresses [63,64,65]. In line with the considerable overlap in Mo gene expression patterns between leaf 4 DPI and root datasets, the majority of genes found upregulated in Bd roots (80.9%) are also detected upregulated in leaves at 4 DPI, compared to 18% shared with the 2 DPI sample. Altogether, these results highlight differences in the upregulation of specific protein family members depending on the infected tissue and fungal lifestyle, while confirming the relevance of these families in response to Mo infections.

Candidate Bd miRNA-generating loci were identified with Shortstack from both control and infected filtered sRNA datasets. Given that the program-assigned cluster IDs are specific to the dataset analyzed and are not comparable between different files, we compared clusters based on their genomic coordinates, and further analyzed loci from the infected sample that had higher RPM (reads per million) than in control, or that were found exclusively in the presence of Mo. This resulted in the identification of 12 miRNA stem loop precursor sequences and 17 mature miRNAs across setups.

Specifically, we identified five mature miRNAs belonging to the MIR156 family, shown to be induced by *F. oxysporum* in *Persicaria minor* [66] and known to be induced by environmental stress, resulting in the cleavage of a SPL (SQUAMOSA promoter binding protein-like) transcription factor and overall modulation of anthocyanin biosynthesis [34,67]. Bd-MIR529, also predicted to target this gene, was found upregulated at 4 DPI in our datasets. Another miRNA involved in the plant response to abiotic stress is miR159b, found upregulated during Mo leaf infection both at 2 and 4 DPI, and known to target two MYB transcription factors in cucumber (*CsGAMYB1* and *CsMYB29*-*like*), involved in ABA signaling [68]. Finally, four additional miRNA families were detected as induced by Mo infection (MIR9484, miR9481b, MIR531 and miR7723a).

To establish the origin of the sRNA reads detected in the different leaf and root setups of the Mo--–Bd interaction, sRNAseq datasets from infected samples were aligned to both the Bd 21-3 and the Mo 70-15 genome. Only reads aligning without mismatches to Mo and with at least two mismatches to Bd were assigned to the fungus and vice-versa, only reads aligning without mismatches to Bd and with at least two mismatches to Mo were assigned to the plant. As expected from the low amount of Mo in infected samples from leaves at 2 DPI, most of the reads were assigned to Bd, whereas higher levels of Mo reads were detected at 4 DPI (leaf) consistent with proliferating infection. All assigned reads were then filtered based on their read counts to select only reads either induced or upregulated in the datasets of infected tissues compared to uninfected tissues and axenic mycelia. We noted that most of the reads (>50%) found in infected samples are specific and are not detected in healthy tissues and axenic culture, showing that sRNA production both in the plant and the fungus is strongly responsive to infection. It follows that sRNA datasets from healthy plants and axenic fungal culture do not represent the full diversity of infection-related sRNA communities. As an additional step, we selected for sRNAs that were not aligning to the coding sequences of the organism of origin. The reasoning behind this filtering step is that we avoided accidental mRNA degradation to be kept as candidate ck-sRNAs, and more important, removed the sRNA sequences more likely to play an endogenous role. Given that the size distribution of upregulated/induced sRNA reads did not show variation in peaks compared to the total sRNA reads, we decided to select 20–22 nt sRNAs (canonical length range for PTGS) for further analysis. Target prediction was carried out with psRNATarget, a web-based prediction software specifically designed for plant sRNA investigations, which allowed for the identification of complementary mRNA sequences in the interacting organism. In PTGS, sRNAs are loaded onto AGO proteins, which guide them towards a complementary mRNA sequence that will then be degraded or sequestered, resulting in reduced levels of the encoded protein. Knowing the expression levels of the predicted targets from the same biological samples used for the sRNA sequencing, we proceeded with further selection of candidate ck-sRNAs based on the significant downregulation of their mRNA targets. Most of the predicted Mo sRNA effectors in the 2 DPI leaf (biotrophic phase) and root samples did not pass this filtering step, as their predicted targets were either upregulated or had comparable expression levels in the corresponding control datasets. There are a few possible explanations as to why the potential targets would not be significantly downregulated in our mRNAseq datasets, including: (i.) the sRNA has not yet been transported throughout the tissue, so the downregulation occurs only at the penetration site, where the fungus is physically interacting with the plant cells, and that is masked by the upregulation in distal parts of the tissue; (ii.) the target mRNA is not cleaved, but its translation is inhibited by the RISC complex acting as a physical barrier, in which case the measurable effect would not be at the mRNA level but only at the protein level; and (iii.) the target is indeed cleaved, but concurrently with the downregulating effect of the sRNA, there is a stronger endogenous upregulation of the gene, leading to either similar levels of mRNA as the control, or even higher.

Comparison of lists with predicted fungal mRNA targets of Bd-sRNAs between the different setups highlights substantial target conservation between the leaf biotrophic and necrotrophic phases, with 140 predicted shared Mo targets between the two, and 42 Mo targets conserved among all three setups. Subjecting the shortlisted predicted fungal target genes to a PHI-base survey for mutations in Mo with lethal or detrimental outcome, we found a clear indication for a reduction in pathogenicity or loss of virulence in respective KO mutants, including *MoATG17* (MGG_07667), an autophagy-related protein whose KO impaired appressorium formation and function, resulting in a complete lack of disease symptoms on rice leaves [45]. Similarly, *MoATG1* (MGG_06393) was also predicted to be targeted by Bd sRNAs and downregulated at 2 DPI, with *Mgatg1* mutant reported by Liu et al. to be unable to infect rice and barley leaves [69]. Additionally, we predicted sRNAs targeting the avirulence effector *AvrPiz-t* (MGG_09055). AvrPiz-t suppresses rice PTI signaling pathway by targeting the E3 ubiquitin ligase APIP6 and suppressing its ligase activity, resulting in reduced flg22-induced ROS generation and overall enhanced susceptibility in vivo [70].

Interestingly, we detected *Calpain-9* to be targeted in all three datasets and significantly downregulated in the foliar ones, both at 2 and 4 DPI. While downregulation in the root setup did not reach a significant padj value, targeting of this transcript would match the finding in the cotton–*V. dahliae* pathosystem, where the host was found to be expressing and exporting miRNAs to the infecting fungus to inhibit its virulence via the targeting of *Clp-1* [39].

Additional predicted targets included integral membrane proteins (MGG_04378T0, MGG_04935T0), ergosterol biosynthesis enzymes (ERG6, MGG_10568T0; ERG26, MGG_04938T0) and fungal cell wall related genes, such as *GPI-anchored cell wall beta-1,3-endoglucanase EglC* (MGG_10400T0, targeted and significantly downregulated in all three datasets), *cell wall protein* MGG_09460T0 (targeted and significantly downregulated in both foliar samples), *chitin deacetylase* (MGG_08774T0) and *chitinase 1* (MGG_08054T0). Targeting fungal membrane components and ergosterol homeostasis has already been proven to be a successful strategy in crop protection against fungal pathogens, both via the application of DMI (sterol demethylation inhibitors) fungicides [71] and with the RNAi-based HIGS (Host-Induced Gene Silencing) and SIGS (Spray Induced Gene Silencing) approaches, where artificial sRNAs are introduced in the plant either via transformation or external application is then transferred to the fungal cells during infection [72,73,74]. It is interesting to observe how the plant appears to have naturally evolved to produce sRNAs potentially able to target fungal essential and pathogenicity related genes. However, Mo is still able to progress its infection both in Bd leaves and roots, and the most likely factors behind this fungal success are the countermeasures it employs in this crosstalk, from the extensive reprogramming of gene expression to the release of effectors and sRNAs.

To substantiate the hypothesis that fungal sRNAs function as effectors to aid the establishment and maintenance of infection, we investigated the role of downregulated Bd targets. Targeting conserved sequences, such as ribosome- and photosynthesis-related ones, and hampering gene expression and biosynthetic processes would prove to be a more effective strategy than specific defense/immunity genes, which are more prone to mutate in the arms race between plants and pathogens.

We confirmed the targeting of a variety of RNA helicases genes, including *BdDCL3b* (BdiBd21-3.2G0305700), identified in our recent work [35] and involved in the preprocessing of sRNA precursor molecules involved in chromatin modification [75]. Specific plant targets included gene families involved in the plant response to both biotic and abiotic stresses, including hormone responsive proteins (BdiBd21-3.5G0286800.1, BdiBd21-3.4G0347500.1), transcription factors such as members of the GATA bHLH families (BdiBd21-3.1G0507400.1, BdiBd21-3.1G0170300.1), peroxidases (BdiBd21-3.3G0739100.1, BdiBd21-3.1G0796400.1, BdiBd21-3.3G0559700.1), disease resistance proteins (BdiBd21-3.2G0771500.1, BdiBd21-3.3G0396200.1) and ABC transporters (BdiBd21-3.5G0309700.1, BdiBd21-3.4G0207200.1, BdiBd21-3.2G0019400.1) [61,63,76,77] Interestingly, *Brachypodium* aquaporins (BdiBd21-3.5G0237900.1) were also downregulated and predicted targets of Mo sRNAs during the infection, consistent with the knowledge that aquaporins play a role in the interaction between plants and microbial pathogens, most likely by modulating both H_2_O availability and transport of ROS [78].

Altogether, the prediction of Mo ck-sRNAs and corresponding Bd targets involved in the plant response to biotic and abiotic stress paves the way for further validation of predicted sRNA/mRNA interactions, including: (i.) proof of target cleavage via RACE or degradome sequencing; (ii.) verification of sRNA–target interaction in transient expression systems such as leaves of *Nicotiana benthamiana*; (iii.) mutational KO strategies of predicted target genes and/or precursor loci of predicted ckRNAs; and (iv.) detection of direct association of sRNAs or their target mRNA with the respective fungal or plant AGO1-like protein by immunopurification techniques [79,80].

## 4. Materials and Methods

### 4.1. AGO and DCL Protein Analysis and 3D Structure Modeling

Known ARGONAUTE and DICER-like protein sequences were downloaded from the NCBI database and analyzed following the workflow utilized for *B. distachyon* AGOs and DCLs [35]. The phylogenetic analysis and tree rendering were done by the Phylogeny.fr web server [81]. Multiple sequence alignment (MSA) of AGO PIWI domains was done using Clustal Omega [82,83] and visualized with the Mview multiple alignment viewer [84]. Protein domains were identified by using Simple Modular Architecture Research Tool (SMART) including PFAM domains in the search [85,86] and visualized with the Illustrator for Biological Sequences (IBS) online illustrator [87]. Prediction of protein location was done using the plant subcellular localization integrative predictor (PSI) [88], and prediction of the interactome was done using the STRING database [89], excluding text mining as indication of putative interaction. Finally, AGOs and DCLs amino acid (aa) sequences were utilized for predicting the proteins’ 3D structure utilizing SWISS-MODEL [90]. Predictions were selected for further validations based on the GMQE and QMEAN Z-score values [91]. PROCHECK [92,93] and WHATCHECK [94] were used to check the stereochemical quality of the selected structures and calculate the Ramachandran Z-score [95]. Open-Source PyMOL (The Py-MOL Molecular Graphics System Version 2.4.0a0) was used for visualization of the predicted structures [96].

### 4.2. Mo Mutants Cultivation and Inoculation

The *Magnaporthe oryzae* wild type Mo 70-15 and mutants Δ*moago1*, Δ*moago2*, Δ*moago3*, Δmodcl1, Δ*modcl2* and Δ*modcl1/2* obtained from N. Donofrio, Newark, NJ, USA, were grown as described [12]. Conidia germination and appressoria development was assessed by incubating conidial suspension (2 × 103 spores mL^–1^) in distilled water on poly-L-lysine coated glass slides (Sigma-Aldrich, St. Louis, MO, USA) in a damp chamber at room temperature for 24 h and examined via inverted microscopy. Fungal stock was prepared on oatmeal agar (OMA) in a regime of 26 °C/24 °C (day/night cycle) and a light intensity of 70 μmol m^−2^ s^−1^ photon flux density. Seeds of *Brachypodium distachyon* cv. ’Bd21-3′ [97] were germinated in soil (Fruhstorfer Erde Typ T) and cultivated in a growth chamber at 22 °C/18 °C (day/night cycle) with 60% relative humidity and a photoperiod of 240 μmol m^−2^ s^−1^ photon flux density. Three methods were utilized to assess disease progression and phenotype of the mutants on leaves: (i.) spray infection of whole seedlings on three-week-old Bd plants with Mo conidia suspension of 120 × 103 spores mL^−1^ in 0.002% Tween20 and assayed on the second youngest leaf; (ii.) spray infection of second youngest detached leaves of three-week-old Bd plants with 250 μL *Magnaporthe oryzae* conidia suspension 50 × 103 spores mL^−1^ in 0.002% Tween20; (iii.) drop inoculation on second youngest detached leaves of three-week-old Bd with 10 μL of conidia solution (50,000 conidia/mL in 0.002% Tween water) on 1% agar plates. Control leaves/seedlings were mock-inoculated with 0.002% Tween water in all setups. Leaves were kept at 16 h light (160 μmol m^−2^ s^−1^)/8 h dark cycle at 22 °C/18 °C. Score disease progression and analysis of the necrotic spots was assayed 5 DPI via ImageJ software [98]. For root inoculation, sterilized seeds (3% NaClO for 15 min, followed by three times 5 min washes in sterile water) of Bd21-3 were vernalized in the dark at 4 °C for two days on half strength MS medium [99] and then moved to a 16 h light (160 μmol m^−2^ s^−1^)/8 h dark cycle at 22 °C/18 °C. Roots of one-week-old seedlings were dip-inoculated in 1 mL of conidia solution (125,000 conidia/mL in 0.002% Tween water) for 3 h and transplanted in a (2:1) mixture of vermiculite (Deutsche Vermiculite GmbH, Sprockhövel, Germany) and Oil-Dri (Damolin, Mettmann, Germany). Control roots were mock-inoculated with 1 mL of 0.002% Tween water solution. Quantification of Mo DNA presence in roots was performed at 5 DPI by quantitative PCR based on the fungal actin (*MoActin*). The experiment was repeated two times, each time with *n* = 10 roots per experimental group.

The *Magnaporthe oryzae* wild type Mo 70-15 and mutants Δ*moago1*, Δ*moago2*, Δ*moago3*, Δmodcl1, Δ*modcl2* and Δ*modcl1/2* obtained from N. Donofrio, Newark, NJ, USA, were grown as described [12]. Conidia germination and appressoria development was assessed by incubating conidial suspension (2 × 103 spores mL^–1^) in distilled water on poly-L-lysine coated glass slides (Sigma-Aldrich, St. Louis, MO, USA) in a damp chamber at room temperature for 24 h and examined via inverted microscopy. Fungal stock was prepared on oatmeal agar (OMA) in a regime of 26 °C/24 °C (day/night cycle) and a light intensity of 70 μmol m^−2^ s^−1^ photon flux density. Seeds of *Brachypodium distachyon* cv. ’Bd21-3′ [97] were germinated in soil (Fruhstorfer Erde Typ T) and cultivated in a growth chamber at 22 °C/18 °C (day/night cycle) with 60% relative humidity and a photoperiod of 240 μmol m^−2^ s^−1^ photon flux density. Three methods were utilized to assess disease progression and phenotype of the mutants on leaves: (i.) spray infection of whole seedlings on three-week-old Bd plants with Mo conidia suspension of 120 × 103 spores mL^−1^ in 0.002% Tween20 and assayed on the second youngest leaf; (ii.) spray infection of second youngest detached leaves of three-week-old Bd plants with 250 μL *Magnaporthe oryzae* conidia suspension 50 × 103 spores mL^−1^ in 0.002% Tween20; (iii.) drop inoculation on second youngest detached leaves of three-week-old Bd with 10 μL of conidia solution (50,000 conidia/mL in 0.002% Tween water) on 1% agar plates. Control leaves/seedlings were mock-inoculated with 0.002% Tween water in all setups. Leaves were kept at 16 h light (160 μmol m^−2^ s^−1^)/8 h dark cycle at 22 °C/18 °C. Score disease progression and analysis of the necrotic spots was assayed 5 DPI via ImageJ software [98]. For root inoculation, sterilized seeds (3% NaClO for 15 min, followed by three times 5 min washes in sterile water) of Bd21-3 were vernalized in the dark at 4 °C for two days on half strength MS medium [99] and then moved to a 16 h light (160 μmol m^−2^ s^−1^)/8 h dark cycle at 22 °C/18 °C. Roots of one-week-old seedlings were dip-inoculated in 1 mL of conidia solution (125,000 conidia/mL in 0.002% Tween water) for 3 h and transplanted in a (2:1) mixture of vermiculite (Deutsche Vermiculite GmbH, Sprockhövel, Germany) and Oil-Dri (Damolin, Mettmann, Germany). Control roots were mock-inoculated with 1 mL of 0.002% Tween water solution. Quantification of Mo DNA presence in roots was performed at 5 DPI by quantitative PCR based on the fungal actin (*MoActin*). The experiment was repeated two times, each time with *n* = 10 roots per experimental group.

The *Magnaporthe oryzae* wild type Mo 70-15 and mutants Δ*moago1*, Δ*moago2*, Δ*moago3*, Δmodcl1, Δ*modcl2* and Δ*modcl1/2* obtained from N. Donofrio, Newark, NJ, USA, were grown as described [12]. Conidia germination and appressoria development was assessed by incubating conidial suspension (2 × 103 spores mL^–1^) in distilled water on poly-L-lysine coated glass slides (Sigma-Aldrich, St. Louis, MO, USA) in a damp chamber at room temperature for 24 h and examined via inverted microscopy. Fungal stock was prepared on oatmeal agar (OMA) in a regime of 26 °C/24 °C (day/night cycle) and a light intensity of 70 μmol m^−2^ s^−1^ photon flux density. Seeds of *Brachypodium distachyon* cv. ’Bd21-3′ [97] were germinated in soil (Fruhstorfer Erde Typ T) and cultivated in a growth chamber at 22 °C/18 °C (day/night cycle) with 60% relative humidity and a photoperiod of 240 μmol m^−2^ s^−1^ photon flux density. Three methods were utilized to assess disease progression and phenotype of the mutants on leaves: (i.) spray infection of whole seedlings on three-week-old Bd plants with Mo conidia suspension of 120 × 103 spores mL^−1^ in 0.002% Tween20 and assayed on the second youngest leaf; (ii.) spray infection of second youngest detached leaves of three-week-old Bd plants with 250 μL *Magnaporthe oryzae* conidia suspension 50 × 103 spores mL^−1^ in 0.002% Tween20; (iii.) drop inoculation on second youngest detached leaves of three-week-old Bd with 10 μL of conidia solution (50,000 conidia/mL in 0.002% Tween water) on 1% agar plates. Control leaves/seedlings were mock-inoculated with 0.002% Tween water in all setups. Leaves were kept at 16 h light (160 μmol m^−2^ s^−1^)/8 h dark cycle at 22 °C/18 °C. Score disease progression and analysis of the necrotic spots was assayed 5 DPI via ImageJ software [98]. For root inoculation, sterilized seeds (3% NaClO for 15 min, followed by three times 5 min washes in sterile water) of Bd21-3 were vernalized in the dark at 4 °C for two days on half strength MS medium [99] and then moved to a 16 h light (160 μmol m^−2^ s^−1^)/8 h dark cycle at 22 °C/18 °C. Roots of one-week-old seedlings were dip-inoculated in 1 mL of conidia solution (125,000 conidia/mL in 0.002% Tween water) for 3 h and transplanted in a (2:1) mixture of vermiculite (Deutsche Vermiculite GmbH, Sprockhövel, Germany) and Oil-Dri (Damolin, Mettmann, Germany). Control roots were mock-inoculated with 1 mL of 0.002% Tween water solution. Quantification of Mo DNA presence in roots was performed at 5 DPI by quantitative PCR based on the fungal actin (*MoActin*). The experiment was repeated two times, each time with *n* = 10 roots per experimental group.

### 4.3. Sample Preparation from Mo–Bd Interaction Sequencing

Mo was grown on oatmeal agar (OMA) for two weeks at 26 °C with 16 h light/8 h dark cycles both for sampling of mycelium and conidia production. Samples from axenic cultures were collected by scraping a mixture of mycelia and spores from three plates, followed by immediate freezing in liquid nitrogen. For root inoculation, sterilized seeds (3% NaClO for 15 min, followed by three times 5 min washes in sterile water) of Bd21-3 were vernalized in the dark at 4 °C for two days on half strength MS medium and then moved to a 16 h light (160 μmol m^−2^ s^−1^)/8 h dark cycle at 22 °C/18 °C. Roots of one-week-old seedlings were dip-inoculated in 1 mL of conidia solution (250,000 conidia/mL in 0.002% Tween water) for 3 h, transplanted in a (2:1) mixture of vermiculite (Deutsche Vermiculite GmbH, Sprockhövel, Germany) and Oil-Dri (Damolin, Mettmann, Germany) and grown for an additional 4 days before harvesting. Control roots were mock-inoculated with 1 mL of Tween water solution. For leaf inoculation, third leaves of three-week-old Bd were detached and drop-inoculated with 10 μL of conidia solution (50,000 conidia/mL in 0.002% Tween water) on 1% agar plates. Control leaves were mock-inoculated with Tween water. Leaves were kept at 6 h light (160 μmol m^−2^ s^−1^)/8 h dark cycle at 22 °C/18 °C and collected for sequencing at 2 days post inoculation (DPI) and 4 DPI.

### 4.4. RNA Extraction, Library Preparation and Sequencing

Three roots or two leaves, respectively, were pooled per sample for RNA extraction and for each condition three pooled biological samples were prepared. Frozen tissue stored at −80 °C was ground in liquid nitrogen using mortar and pestle. Total RNA was isolated with ZymoBIOMICS TM RNA Mini Kit (Zymo Research, Irvine, CA, USA) according to the manufacturer’s instructions. Quantity and integrity of the RNA were assessed with DropSense16/Xpose (BIOKÉ, Leiden, Netherlands) and Bioanalyzer 2100 (Agilent, Santa Clara, CA, USA), respectively. Purification of small and large RNAs into separate fractions was carried out using RNA Clean & Concentrator TM -5 (Zymo Research, Irvine, CA, USA) and concentration and quality of the fractions were checked again, using the Agilent 2100 Bioanalyzer Pico Chip and the Qubit fluorometer (Invitrogen, Carlsbad, CA, USA) for the sRNA fraction. A total of 50 ng of sRNA (17 to 200 nt) were used for cDNA library preparation with TruSeq^®^ Small RNA Library Prep (Illumina, San Diego, CA, USA) and 1.5 μg of large RNA were used for cDNA library preparation with TruSeq^®^ Stranded mRNA (Illumina, San Diego, CA, USA). Constructed cDNA libraries of sRNAs were further size selected with BluePippin (Sage Science, Beverly, MA, USA) for fragments between 140 and 160 nt (15–35 nt without adapters). Quality of polyA mRNA libraries was assessed using the Fragment AnalyzerTM Automated CE System (Advanced Analytical Technologies, Heidelberg, Germany). The Illumina HiSeq1500 sequencing platform was used to sequence the Illumina TruSeq^®^ Small RNA libraries single end with 35 nt read length and the Illumina TruSeq^®^ Stranded mRNA libraries (paired-end [PE] sequencing, 70 nt) of all samples.

### 4.5. Transcriptome Analysis

Paired end sequenced cDNA reads of Illumina TruSeq^®^ Stranded mRNA libraries were analyzed through the quality check in FastQC and alignment in the junction mapper HISAT2 [100]. *Magnaporthe oryzae* MG8 release 38 [101] and *Brachypodium distachyon* Bd21-3 v1.1 (DOE-JGI, http://phytozome.jgi.doe.gov/) assemblies were utilized throughout this study as references. Htseq-count [102] and DESeq2 [103] were then used for read counting and differential gene expression calling (DGE) between the infected and control sample genes and to generate volcano plots. Heatmaps for selected DEGs were obtained with the pheatmap package for R [104]. Gene Ontology Enrichment (GOE) analysis on DEGs was done with AgriGO v2 [105]. Gene descriptions were integrated from the organism genome assembly, ENSEMBL Biomart, Phytozome and Blast2GO [106].

### 4.6. sRNA Analysis, Prediction of Endo- and Cross-Kingdom sRNA

The single end sequenced cDNA reads of Illumina TruSeq^®^ Small RNA libraries were analyzed starting with quality check with FastQC [107] and trimming of adapter artifacts with cutadapt [108]. The alignment of the reads to reference genomes and transcriptomes of Bd and Mo was done using the short read aligner Bowtie [109]. Reads with a 100% alignment to the genome of the organism of origin were selected, alongside the reads with at least two mismatches in the alignment to the target organism genome. Venn diagrams for sRNA and target overlaps were obtained with the VennDiagram package for R [110].

To identify interaction-related Bd sRNAs with endogenous function, both infected and control datasets were analyzed with ShortStack [111] to identify potential miRNA generating loci. Genomic coordinates and corresponding reads per million (RPM) of the identified clusters were compared between infected and control datasets to select clusters exclusively present or increased during infection. Both potential precursors and mature miRNAs deriving from these clusters were compared to known miRNA sequences, obtained from miRBase [112]. The structure of miRNA generating clusters was visualized with strucVis (version 0.4, Michael J. Axtell).

Bioinformatics analysis of candidate ck-sRNAs was done as described in Zanini et al. [43]. Only sRNA reads of 20–22 nt originating from non-coding regions and with a higher count in the organism of origin control datasets compared to the infected ones were analyzed further for ck-sRNA effector identification by the target prediction software psRNATarget used with customized settings [113].

Expression levels obtained for each gene were used as confirmation of downregulation of predicted targets from the psRNATarget software. PHI-base, a collection of experimentally verified pathogenicity/virulence genes from fungal and microbial pathogens [114], was used to gather information regarding phenotype and virulence of fungal mutants carrying a mutation in the identified Mo gene targets.

## 5. Conclusions

In the present work, we analyzed and characterized the interaction between *Brachypodium distachyon* and *Magnaporthe oryzae* at different fungal lifestyles and infection sites, both from a transcriptomic and sRNA expression profiles’ point of view. The pathosystem has been studied as a model for the effect of blast disease on staple crops leaves (e.g., rice, wheat and barley), owing to Bd’s short maturation phase, smaller genome and space-saving production [11,12,14]. In addition to foliar infections, we also established and characterized the interaction and responses to Bd root colonization by Mo. Additional to the confirmation of the extensive reprogramming in both organisms throughout the interaction, our results support the possibility that major staple crops co-evolved mechanisms of RNA-based communication with their microbial pathogens. Based on concomitant deep sequencing of mRNA and sRNA fractions, our work provides the first indication of both plant and fungal sRNAs involvement in the communication between *Magnaporthe oryzae* with the model grass *Brachypodium distachyon*, further supporting the theory of ckRNAi participation in plant–pathogen interactions. Interestingly, sRNAs induced during infection setups show only partial overlap both among the different tissues (leaves, roots) and the different infection phases (leaf: biotrophic, necrotrophic), raising the possibility that ckRNAi in a given host–pathogen interaction exhibits tissue- and lifestyle-specificity.

## Figures and Tables

**Table 1:** Total numbers of significantly (padj < 0.05) upregulated or downregulated genes in the *B. distachyon*–*M. oryzae* interaction.

Setup	Total Mo Genes (Up)	Total Mo Genes (Down)	Total Bd Genes (Up)	Total Bd Genes (Down)
Leaf 2 DPI	1041	1094	224	9
Leaf 4 DPI	1710	1476	3023	1955
Root	673	327	89	39

Overview of DESeq2 results. DESeq2 test for differential expression based on negative binomial distribution. Abbreviation: DPI = days post inoculation.

**Table 2:** Selected defense-related Bd DEGs (padj < 0.05) during infection of leaves and roots by *M. oryzae*.

Gene	Description	Log_2_FC- Leaf 2 DPI	Log_2_FC- Leaf 4 DPI	Log_2_FC- Root
BdiBd21-3.3G0465100	ABC transporter a family member 2-like	1.15	3.38	0.79
BdiBd21-3.3G0464900	ABC transporter a family member 7-like		2.64	0.71
BdiBd21-3.2G0605400	ABC transporter b family member 4-like	2.15	5.08	
BdiBd21-3.2G0550500	Pleiotropic drug resistance protein 3-like	1.78	6.82	1.51
BdiBd21-3.5G0159100	Anthranilate synthase component ii	0.76	2.75	0.88
BdiBd21-3.3G0344500	Chitinase 1		2.70	
BdiBd21-3.2G0371800	Cytochrome p450 71c4		1.32	2.12
BdiBd21-3.1G0952300	Disease resistance response		2.07	
BdiBd21-3.3G0144800	Protein kinase xa21	1.77	2.41	1.24
BdiBd21-3.2G0545400	LRR receptor-like serine threonine-protein kinase gso1		1.59	1.79
BdiBd21-3.2G0632400	Receptor-like protein kinase hsl1-like		3.15	
BdiBd21-3.1G0713100	Proton-coupled amino acid transporter 3-like	0.85	1.97	0.97
BdiBd21-3.2G0114800	Pathogenesis related protein	1.34	1.25	
BdiBd21-3.1G0165000	Pathogenesis-related protein 1	6.06	8.19	
BdiBd21-3.4G0068000	Pathogenesis-related protein 10		3.47	3.75
BdiBd21-3.4G0043000	Pathogenesis-related protein 5		2.91	
BdiBd21-3.1G0772600	Pathogenesis-related protein class i		5.29	4.47
BdiBd21-3.1G0772700	Pathogenesis-related protein prb1-2-like		3.41	6.42
BdiBd21-3.3G0422200	PR3-like 1		2.63	
BdiBd21-3.3G0639500	PR3-like 2		3.96	
BdiBd21-3.4G0025400	PR5-like		1.37	
BdiBd21-3.2G0600500	PR5-like		2.28	
BdiBd21-3.1G0875700	Pathogenesis-related protein Bet v I family		2.14	
BdiBd21-3.1G0054700	NAC1 transcription factor		2.49	
BdiBd21-3.3G0652700	MYB-related protein myb4-like		3.33	
BdiBd21-3.2G0688100	Probable WRKY transcription factor 33-like		2.69	
BdiBd21-3.2G0615100	Probable WRKY transcription factor 51-like	1.45	3.44	
BdiBd21-3.3G0669400	Ethylene-responsive transcription factor 1a		2.16	
BdiBd21-3.4G0171000	Multicopper oxidase family expressed	4.35	9.93	2.35
BdiBd21-3.4G0387000	Cationic peroxidase spc4-like	3.17	3.72	
BdiBd21-3.1G0233900	Peroxidase		2.11	
BdiBd21-3.2G0563800	Secologanin synthase-like	0.57	0.54	1.08

**Table 3:** Selected significantly (padj < 0.05) upregulated pathogenicity-/virulence-related *M. oryzae* genes during *B. distachyon* leaves and roots infection.

Gene Stable ID	Description	Log_2_FC- Leaf 2 DPI	Log_2_FC- Leaf 4 DPI	Log_2_FC- Root
MGG_00750	Cytochrome b-245 heavychain subunit beta	2.23	2.12	2.80
MGG_01081	Peroxin 14/17		1.05	
MGG_01092	Homocitrate synthase	1.35		
MGG_01748	Putative protease		1.28	
MGG_02074	Potassium/sodium efflux P-type ATPase		1.28	2.55
MGG_03356	Ricin B lectin:Parallel beta-helix		7.08	5.06
MGG_04202	MAS3 protein	2.27		2.75
MGG_04212	L-ornithine 5-monooxygenase	2.81	3.46	3.17
MGG_04301	Pwl2 protein (PWL2) gene	8.53		
MGG_04545	Cytochrome c peroxidase, mitochondrial	3.26	0.95	
MGG_06011	S-(Hydroxymethyl)glutathione dehydrogenase		2.53	
MGG_06648	Hsp70 (LHS1) gene	1.25		
MGG_07514	3-oxoacyl-[acyl-carrier-protein] reductase		1.49	
MGG_07971	Calcium-transporting ATPase 1		1.80	
MGG_08315	1-phosphatidylinositol-4,5-bisphosphate phosphodiesterase delta-1	8.83	7.94	
MGG_08409	Cellulose-growth-specific protein		3.78	
MGG_09022	Transmembrane CFEM domain-containing protein	5.41	7.42	7.88
MGG_09559	Autophagy-related protein 9		1.07	
MGG_09956	PRO41 protein	1.93	1.91	2.62
MGG_10097	Intracellular hyphae protein 1	5.42		
MGG_10510	Ribonuclease T2	3.90		
MGG_10730	Potassium/sodium efflux P-type ATPase		4.75	
MGG_11882	Sensor protein zRas		1.60	3.15
MGG_11899	SH3 domain-containing protein	1.93	1.52	
MGG_15370	Metalloproteinase	11.86		
MGG_15972	AVR-pik/pikm/pikp	14.63	6.33	

**Table 4:** *B. distachyon* miRNA identification.

Setup	Cluster Number	miRNA	Cluster RPM	Mature miRNA Name
Leaf 2 DPI	3086	Bdi-miR159b	118.27	miR159b-3p.2
				miR159b-3p.1
	3421	Bdi-MIR531	15.03	MIR531
	6495	Bdi-MIR156b	2.61	miR156b-3p
	7687	Bdi-miR9481b	19.12	miR9481b -5p
				miR9481b-3p
Leaf 4 DPI	7744	Bdi-MIR156h	13.79	MIR156h-5p
	3312	Bdi-MIR159b	184.31	miR159b-3p.1
				miR159b-3p.2
				miR159b-5p.1
				miR159b-5p.2
	3162	Bdi-MIR171d	0.58	MIR171d-3p
	7470	Bdi-MIR529	30.80	MIR529-5p
	2384	Bdi-miR7723a	13.02	miR7723a-3p
	10592	Bdi-MIR156d	8.31	MIR156d-5p
Root	8229	Bdi-MIR156i	2.87	MIR156i-5p
				MIR156i -3p
	5121	Bdi-MIR168	310.27	MIR168-5p
	9081	Bdi-MIR156d	1.15	miR156d-5p
	4330	bdi-MIR9484	3.90	MIR9484

miRNA-generating clusters were identified with Shortstack in all datasets. Genomic coordinates were used to detect and compare the same loci in infected and control samples, and only clusters exclusive or increased in the infected samples were selected. Clusters and mature miRNAs were compared to known miRNA/miRNA precursor from miRBase. Abbreviation: RPM = reads per million.

**Table 5:** Overview of the number of predicted cross-kingdom sRNA candidates (20–22 nt) and their corresponding target mRNAs after target downregulation confirmation (FC < 0, padj < 0.05).

	Setup	Number of sRNA Candidates	Number of sRNA Candidates with Downregulated Targets	Number of Targets Predicted	Number of Targets Downregulated	
Mo sRNAs	Leaf 2 DPI	604	7	25106	5	Bd mRNAs
	Leaf 4 DPI	546	490	25415	1128	
	Root	394	14	17335	10	
Bd sRNAs	Leaf 2 DPI	424	314	4621	484	Mo mRNAs
	Leaf 4 DPI	302	236	4431	527	
	Root	681	263	6730	183	

**Table 6:** Selection of Mo sRNA/Bd mRNA duplexes from different leaf and roots setups.

Setup	sRNA	RPM I	Target	Log_2_FC	Description
Leaf 2 DPI	TTTCGACGCTGCCCTGACTT	31.9	BdiBd21-3.1G0045900.1	−0.49	Bowman–Birk type trypsin inhibitor
	TTTCGACGCTGCCCTGACTT	31.9	BdiBd21-3.4G0610700.1	−1.25	probable apyrase 3
	GGTTATCATCGTCCCAGCCC	15.9	BdiBd21-3.4G0347500.1	−0.70	abscisic stress-ripening protein 3
Leaf 4 DPI	TGGCAGCGGCGCAGGATCTCG	8.5	BdiBd21-3.5G0309700.1	−1.39	ABC transporter B family member 19
	CAATCGTTGTCTGGCATTGA	83.4	BdiBd21-3.4G0207200.1	−0.72	ABC transporter F family member 5
	TTGTGTCCAAGCGTTCTGAAA	13.3	BdiBd21-3.2G0019400.1	−0.88	ABC transporter G family member 7
	TGATTAAGGAGAAGCGGGGG	6.0	BdiBd21-3.5G0286800.1	−0.95	auxin-responsive protein SAUR71
	TCGCTTTGGCGGCGCGCCGGC	27.8	BdiBd21-3.1G0937800.1	−1.15	cytochrome P450 94B3
	TCGCACTTCGCGGCGTTGGCG	16.9	BdiBd21-3.3G0497100.1	−1.11	cytokinin dehydrogenase 11
	AGGGGCTACGATCTTTGAGAA	18.1	BdiBd21-3.3G0787100.1	−1.13	DexH-box ATP-dependent RNA helicase DExH15
	TTTCGAGATTGGAAACGGCT	12.1	BdiBd21-3.2G0305700.1	−0.69	dicer homolog 3b
	TGACGGGATAGGTAAAGAACTA	8.5	BdiBd21-3.2G0095300.1	−0.72	G-type lectin S-receptor-like serine/threonine-protein kinase
	TAGATCGGTTGGTGTCGGGC	109.9	BdiBd21-3.1G0507400.1	−0.66	GATA transcription factor 21
	TGGGCGGCGGTCATTTCGGC	6.0	BdiBd21-3.3G0739100.1	−1.86	peroxidase 1
	AGAAGAATTTCATGCCGGCCAG	8.5	BdiBd21-3.1G0796400.1	−0.71	peroxidase 50
	CCGGCATGAAATTCTTCTCGAA	7.2	BdiBd21-3.1G0093800.1	−0.99	photosystem I reaction center subunit III, chloroplastic
	TAGTAGGGCTGCAAGATCTA	10.9	BdiBd21-3.5G0116500.1	−0.91	photosystem I subunit PsaO
	TAGTTGAGTTCCGCCTGCTG	58.0	BdiBd21-3.1G0118800.1	−0.60	photosystem II D1 precursor processing protein PSB27-H2, chloroplastic
	TGGCTGTGAATTCGGCGAGGG	59.2	BdiBd21-3.5G0237900.1	−0.62	probable aquaporin PIP1-2
	CCAATCGTTGTCTGGCATTGA	105.1	BdiBd21-3.2G0771500.1	−1.33	putative disease resistance protein RGA3
	TTTCGGATAGAGGCACCCAA	10.9	BdiBd21-3.3G0396200.1	−0.54	putative disease resistance protein RGA4
	TATCGTCGCGCAGTTGGTCG	7.2	BdiBd21-3.3G0461000.1	−0.56	RNA exonuclease 4
	TGAGCCGGGGGTATAATCGG	6.0	BdiBd21-3.4G0029600.1	−0.65	stress-induced-phosphoprotein 1
	TGATTCGGCGGCAGGTCTGGC	14.5	BdiBd21-3.1G0170300.1	−1.17	transcription factor bHLH25
	TCGGTTTCGGCTTCTGGGGT	10.9	BdiBd21-3.2G0063400.1	−1.19	transcription factor NIGTH1
	TAAATACCGTCCCGGCAAGG	9.7	BdiBd21-3.4G0018600.1	−0.66	wall-associated receptor kinase 5
	TGACGGAGCTCGGCCTGGAA	45.9	BdiBd21-3.5G0304000.1	−0.90	wound-induced protein
Root	TAGGGTGGCCTGAATTATAGT	10.4	BdiBd21-3.2G0378400.1	−0.74	glycine-rich cell wall structural protein 1
	AGTATTCCGTCGTCGCCGTA	20.7	BdiBd21-3.3G0257600.1	−0.78	xyloglucan endotransglycosylase/hydrolase protein 8
	TGAACCAGCCGTTGAGTAAG	10.4	BdiBd21-3.3G0280200.1	−0.55	fatty acyl-CoA reductase 1

Abbreviations: RPM = reads per million; I = infected sample; FC = fold change.

**Table 7:** Selected *M. oryzae* mRNA targets of predicted cross-kingdom *B. distachyon* sRNAs from leaf and root setups.

Predicted Target			Target ID	Target Description	Log_2_FC		
Leaf 2 DPI	Leaf 4 DPI	Root			Leaf 2 DPI	Leaf 4 DPI	Root
X	X	X	MGG_02127	alcohol oxidase	−5.46	−2.53	−3.11
X	X	X	MGG_02695	cysteine proteinase 1	−4.72	−1.35	−2.46
X	X	X	MGG_06494	D-arabinitol 2-dehydrogenase	−5.52	−1.16	−3.21
X	X	X	MGG_01386	FAD dependent oxidoreductase superfamily protein	−5.19	−2.16	−4.51
X	X	X	MGG_05981	glutamine amidotransferase subunit pdxT	−4.79	−2.34	−4.25
X	X	X	MGG_10400	GPI-anchored cell wall beta−1,3-endoglucanase EglC	−0.99	−2.05	−2.11
X	X	X	MGG_01361	PHO85 cyclin-1	−5.81	−1.27	−2.85
	X		MGG_15576	DNA repair protein rhp51	n.s.	−1.05	n.s.
	X		MGG_03587	essential for mitotic growth 1	n.s.	−0.60	n.s.
X	X		MGG_04345	cytochrome P450 17A1	−4.40	−1.06	n.s.
X		X	MGG_03201	acetyl-coenzyme A synthetase	−1.39	0.86	−1.84
	X		MGG_09950	C2H2 type zinc finger domain-containing protein	n.s.	−1.49	n.s.
	X	X	MGG_16901	ATP-dependent RNA helicase DBP2	n.s.	−1.04	−1.18
X	X	X	MGG_07667	autophagy-related protein 17	−2.45	−1.33	n.s.
X	X	X	MGG_01391	ent-kaurene oxidase	−3.90	−2.44	n.s.
X	X	X	MGG_11962	G-protein coupled receptor	−5.75	−3.42	n.s.
X	X	X	MGG_04378	integral membrane protein	−3.99	−1.04	n.s.
X	X	X	MGG_04935	integral membrane protein	−3.58	−1.40	n.s.
X	X	X	MGG_03123	MATE efflux family protein subfamily	−4.68	−1.11	n.s.
X	X	X	MGG_14872	calpain-9	−4.19	−1.17	n.s.
X	X	X	MGG_09460	cell wall protein	−4.72	−3.92	n.s.
X	X	X	MGG_03186	1,4-alpha-glucan-branching enzyme	−1.52	n.s.	−1.36
X	X	X	MGG_14154	RETRO5, retrotransposons MoTeR1s and MoTeR2	−6.04	−1.15	n.s.
X		X	MGG_06393	serine/threonine-protein kinase ATG1	−3.24	n.s.	n.s.
	X	X	MGG_04938	C-3 sterol dehydrogenase/C-4 decarboxylase	n.s.	−1.85	n.s.
X	X	X	MGG_10568	sterol 24-C-methyltransferase	n.s.	n.s.	−1.24
X		X	MGG_06371	pyruvate dehydrogenase E1 component subunit alpha	n.s.	n.s.	−1.75

X is assigned if the gene is predicted to be targeted by candidate sRNA in the corresponding setup. Abbreviations: FC = fold change; n.s. = not significant.

**Table 8:** Predicted RNAi targets with known infection-impaired phenotypes in the corresponding *M. oryzae* knock-out (KO) mutants.

Transcript ID	Gene ID	Description	Phenotype	Host Species	Reference Phi-Base
MGG_00063T0	AGL1	glycogen debranching enzyme	reduced_virulence	Os_Hv	PHI:3814
MGG_00365T0	MAGB	G alpha protein subunit	reduced_virulence	Os	PHI:83
MGG_00620T0	MoDac	GlcNAc-6-phosphate deacetylase	reduced_virulence	Os	PHI:5471
MGG_01096T0	SGA1	vacuolar glucoamylase	loss_of_pathogenicity	Os_Hv	PHI:2138
MGG_01180T0	MoSCAD3	short-chain specific acyl-CoA dehydrogenase	reduced_virulence	Os_Hv	PHI:8929
MGG_01285T0	Tpc1	sranscription factor for Polarity Control 1	reduced_virulence	Os_Hv	PHI:7317
MGG_01819T0	Gph1	phosphorylase	loss_of_pathogenicity/reduced_virulence	Os_Hv	PHI:2062/3815
MGG_02444T0	MoPLC1	modulator of calcium flux	loss_of_pathogenicity	Os	PHI:2057
MGG_02457T0	RHO2	Rho GTPase	reduced_virulence	Os	PHI:8752
MGG_02884T0	MoFLP1	fasciclin-like protein	reduced_virulence	Os_Hv	PHI:4231
MGG_03148T0	TDG4	trigalactosyldiacylglycerol-4	reduced_virulence	Os_Hv	PHI:3811
MGG_03198T0	TIG1	histone deacetylation	loss_of_pathogenicity	Os_Hv	PHI:2002
MGG_03670T0	SPM1	subtilisin-like proteinase Spm1	reduced_virulence	Os_Hv	PHI:2117
MGG_03860T0	TPS1	trehalose-6-phosphate synthase	loss_of_pathogenicity/reduced_virulence	Os_Hv	PHI:322/1064
MGG_04895T0	ICL1	isocitrate lyase	reduced_virulence	Os	PHI:305
MGG_05344T0	MgSM1	effector	increased_virulence	At	PHI:2118/5540
MGG_06393T0	ATG1	autophagy-related protein 1	loss_of_pathogenicity	Os_Hv	PHI:2035/2069/8612
MGG_07667T0	Moatg17	autophagy-related protein 17	loss_of_pathogenicity	Os_Hv	PHI:2083
MGG_08054T0	MoChi1	chitinase 1	reduced_virulence	Os_Hv	PHI:8753/8806
MGG_08370T0	gel3	1,3-beta-glucanosyltransferase	loss_of_pathogenicity	Os	PHI:6713
MGG_09471T0	NTH1	neutral trehalase	reduced_virulence	Os_Hv	PHI:123/775/794
MGG_10859T0	MoLDS1	heme peroxidase	reduced_virulence	Os	PHI:5189
MGG_11862T0	ABC4	ABC transporter	reduced_virulence/loss_of_pathogenicity	Hv	PHI:1017/2067
MGG_12122T0	MoGSK1	glycogen synthase kinase 1	loss_of_pathogenicity	Os_Hv	PHI:7117
MGG_12814T0	MoAP1	BZIP domain-containing protein	loss_of_pathogenicity	Os_Hv	PHI:2142
MGG_17909T0	ATG3	autophagy-related protein 3	loss_of_pathogenicity	Os	PHI:2071
MGG_05287T0	CON7	transcription factor CON7	loss_of_pathogenicity	Os_Hv	PHI:2039
MGG_09055T0	AvrPiz-t	avrpiz-tgene, effector protein	increased_virulence	Os	PHI:7896

Information derived from Pathogen-Host Interactions Database (PHI-base) search.

## Data Availability

The data presented in this study are openly available in ArrayExpress, accessions E-MTAB-9985 and E-MTAB-9984.

## Supplementary Materials

The following are available online at https://www.mdpi.com/1422-0067/22/2/650/s1, Figure S1: Analysis of MoAGO and MoDCL protein sequences, Figure S2: Multiple sequence alignment of the PIWI domain of MoAGO proteins, Figure S3: Development of appressoria from conidia of Mo wt and RNAi mutants, Figure S4: Phenotypic analysis of Mo wt and RNA interference mutants, Figure S5: Heatmap for Mo and Bd DEG calling with DESeq2, Figure S6: Results of gene ontology enrichment (GOE) analysis for significantly DE Bd genes in the 4 DPI leaf setup, Figure S7: Results of gene ontology enrichment (GOE) analysis for significantly DE Mo genes in the root setup, Figure S8: Visual representation of the identified upregulated Bd clusters (miRNA precursors) structures, Table S1: Domain structures and coordinates of MoAGOs and MoDCLs as detected by SMART+PFAM search, Table S2: MoAGOs and MoDCLs protein localization prediction results by PSI, Table S3: Prediction of protein interactome for MoDCLs and MoAGOs, Table S4: Expression changes of AGOs and DCLs during the interaction of (A) *B. distachyon* and (B) M. oryzae from mRNAseq results, Table S5: Selection of significantly enriched Bd GO terms from 2 DPI, 4 DPI and root Bd DEG datasets, Table S6: Selection of significantly enriched Mo GO terms from 2 DPI, 4 DPI and root Mo DEG datasets, Table S7: Overview of total sRNA and mRNA reads in the *Brachypodium distachyon*–*Magnaporthe oryzae* interaction.
