# Supplementary material for: Comparative Analysis of Transcriptome and sRNAs Expression Patterns in the Brachypodium distachyon—Magnaporthe oryzae Pathosystems
Source: Int J Mol Sci. 2021 Jan 11;22(2):650. doi: 10.3390/ijms22020650 (PMC7826919; doi:10.3390/ijms22020650)
Supplement: Supplementary file 1 [file ijms-22-00650-s001.zip › Zanini_et_al_2020_Supplementary_figures.pdf]

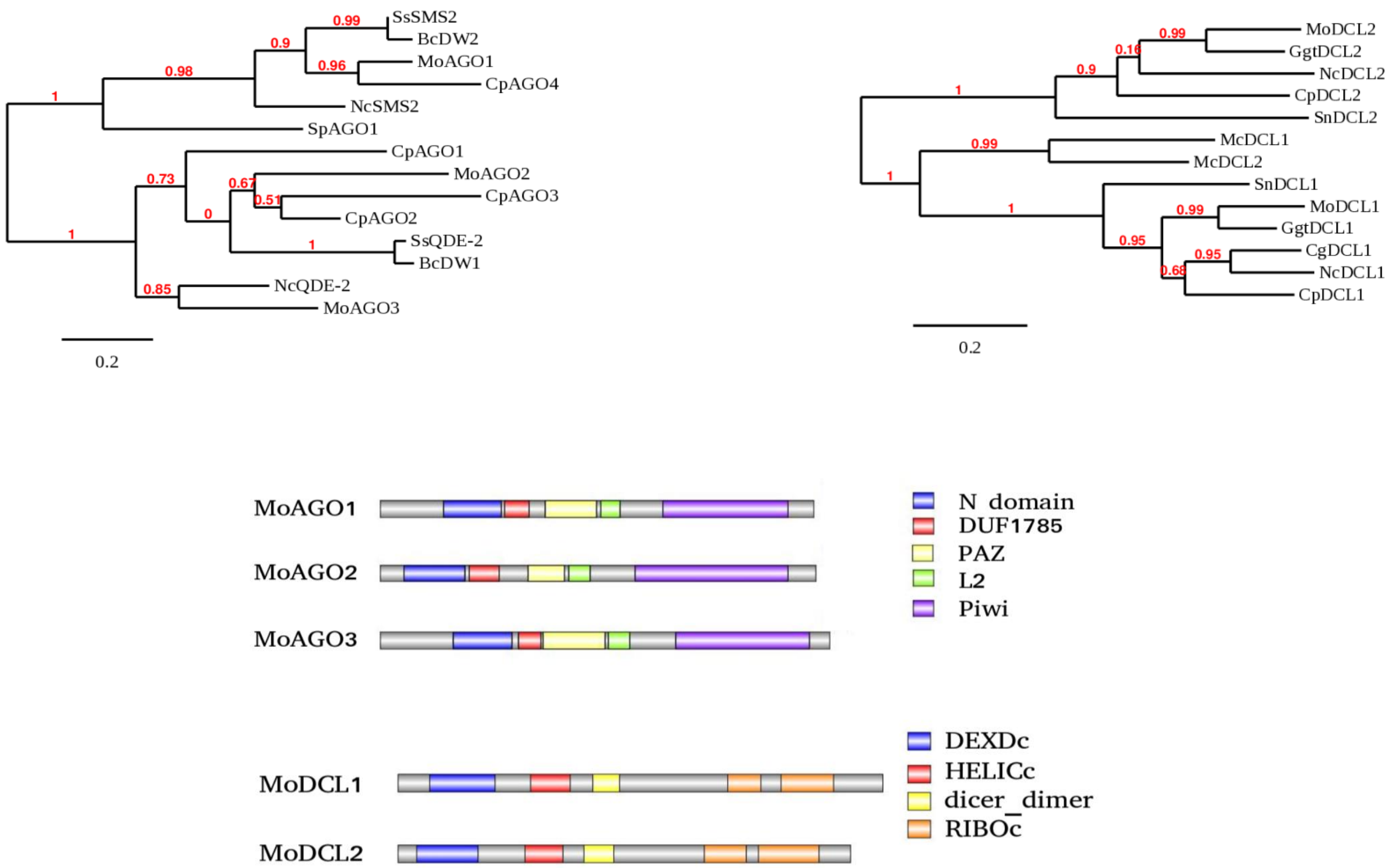

Figure S1. Analysis of MoAGO and MoDCL protein sequences. Phylogram of (A) MoDCL and (B) MoAGO protein sequences. Branch support values are displayed, the scale bar defines the branch length. Ss= Sclerotinia sclerotiorum, Bc= *Botrytis cinerea*, Cp= *Cryphonectria parasitica*, Sp= *Schizosaccharomyces pombe* , Nc= *Neurospora crassa*, Ggt= *Gaeumannomyces graminis var. tritici* Mc= Mucor *circinelloides* Sn= *Parastagonospora nodorum* Cg= *Chaetomium globosum*. Visual representation of domain structure of (C) MoAGO and (D) MoDCL proteins. Domains were identified by SMART and PFAM search and represented with IBS illustrator. Displayed domains of AGOs: N-domain, DUF1785, PAZ, L2, PIWI. Sequence with no domain predicted is colored in grey. Displayed domains of DCLs: DEXDc, HELICc, dicer_dimer and RIBOc.

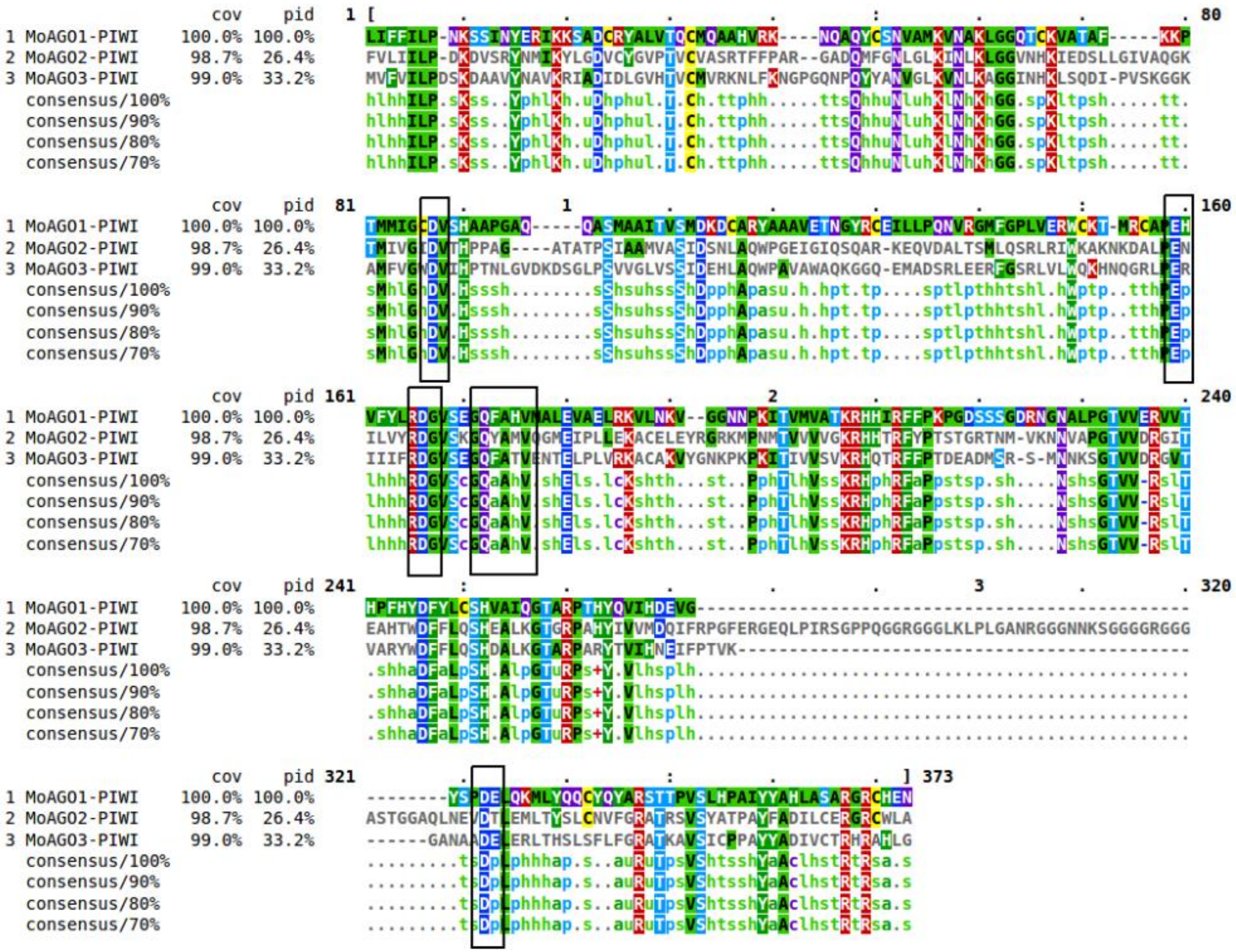

Figure S2. Multiple sequence alignment of the PIWI domain of MoAGO proteins. Sequences were selected based on SMART domain identification, aligned with Clustal Omega and visualized with Mview. The catalytic tetrad DEDD and the QF-V motifs are boxed.

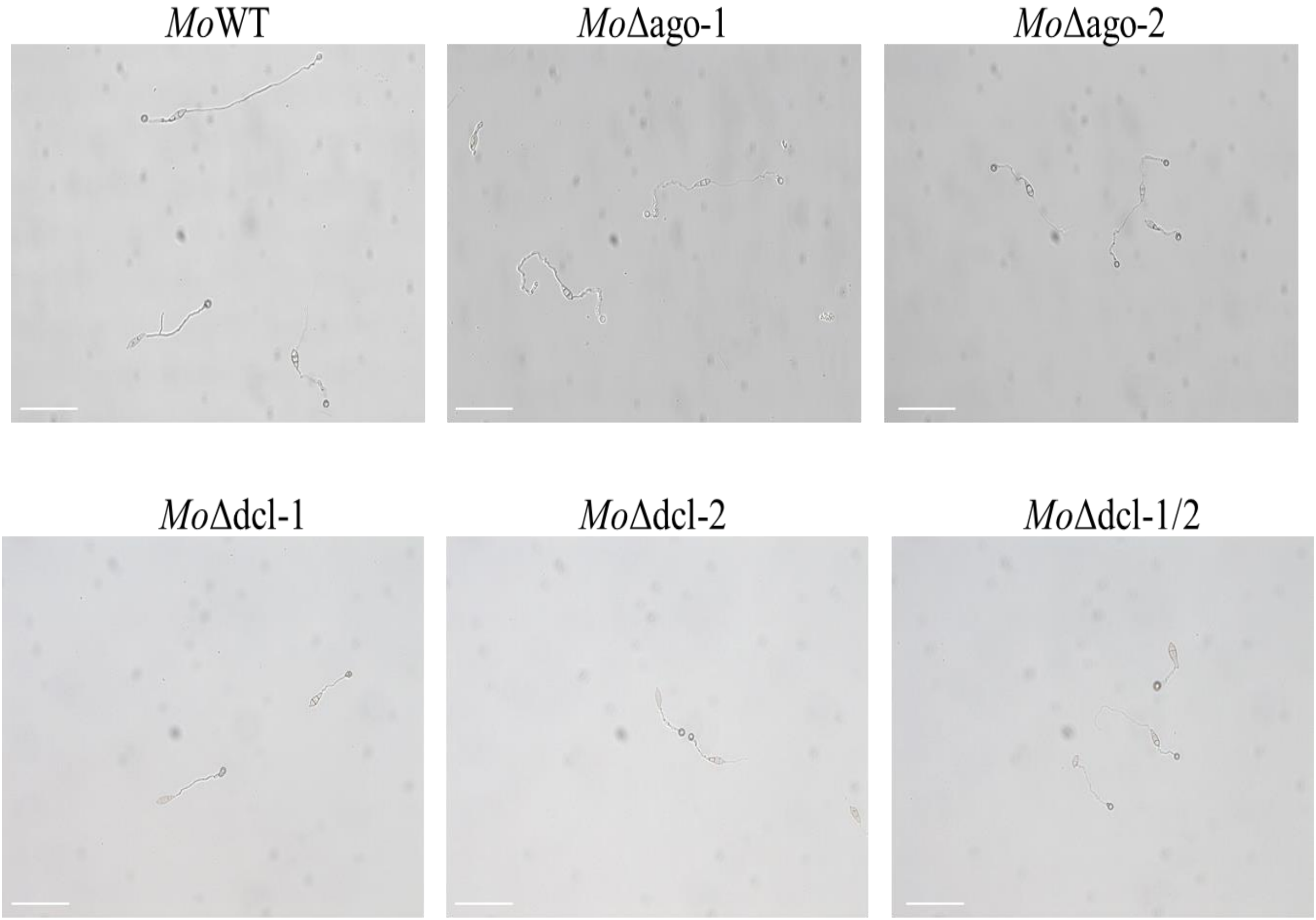

Figure S3. Development of appressoria from conidia of Mo WT and RNAi mutants. Conidial suspensions at 2 × 103 ml−1 of Mo70-15 and mutant strains in distilled water were inoculated onto poly-L-lysine-coated glass coverslips in a damp chamber and examined for appressorium formation after 24 HPI. The experiment was conducted 3 times with similar results. White scale bar = 80μm.

Whole seedling spray

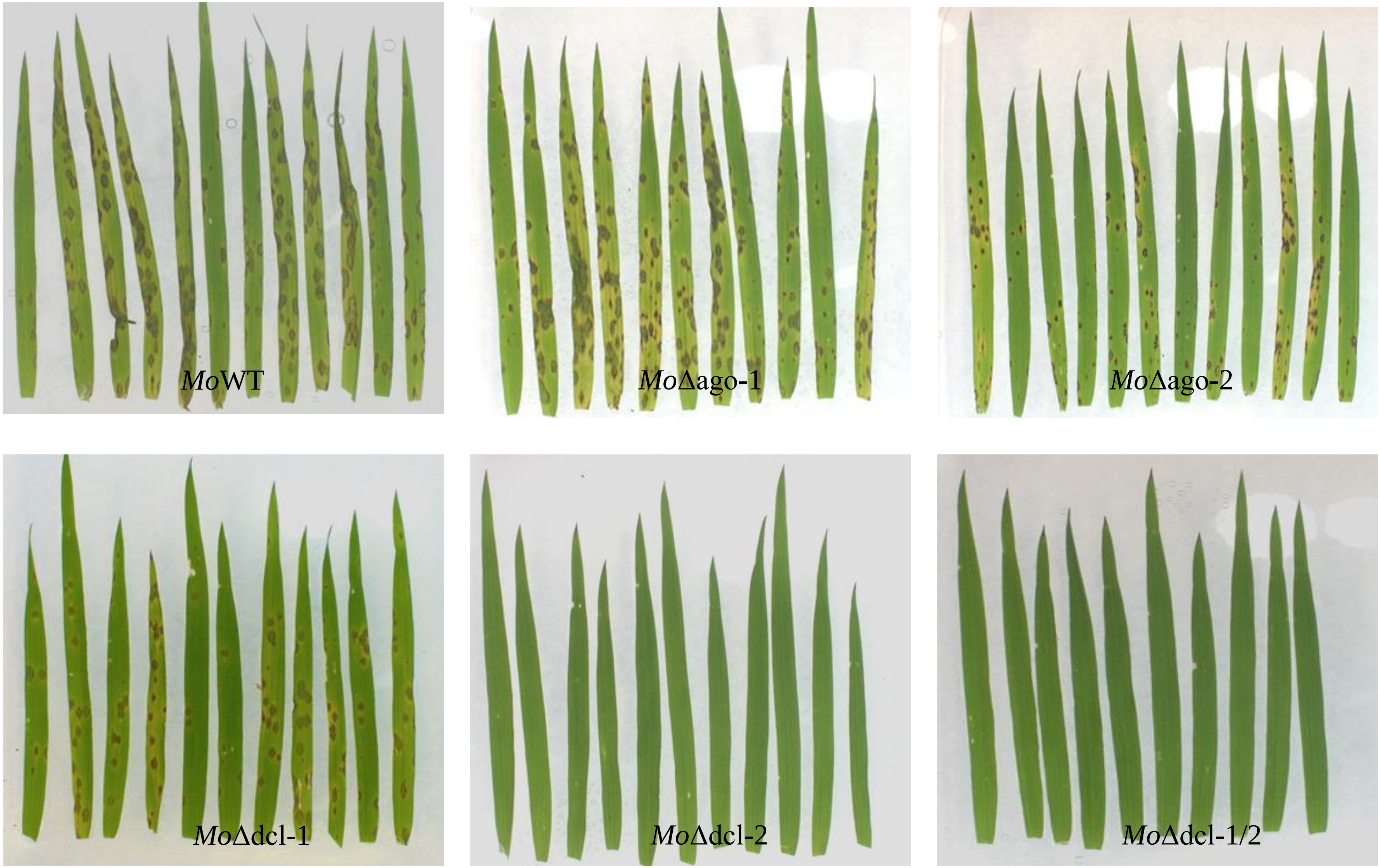

Figure S4. Phenotypic analysis of Mo WT and RNA interference mutants.

Drop inoculation assay

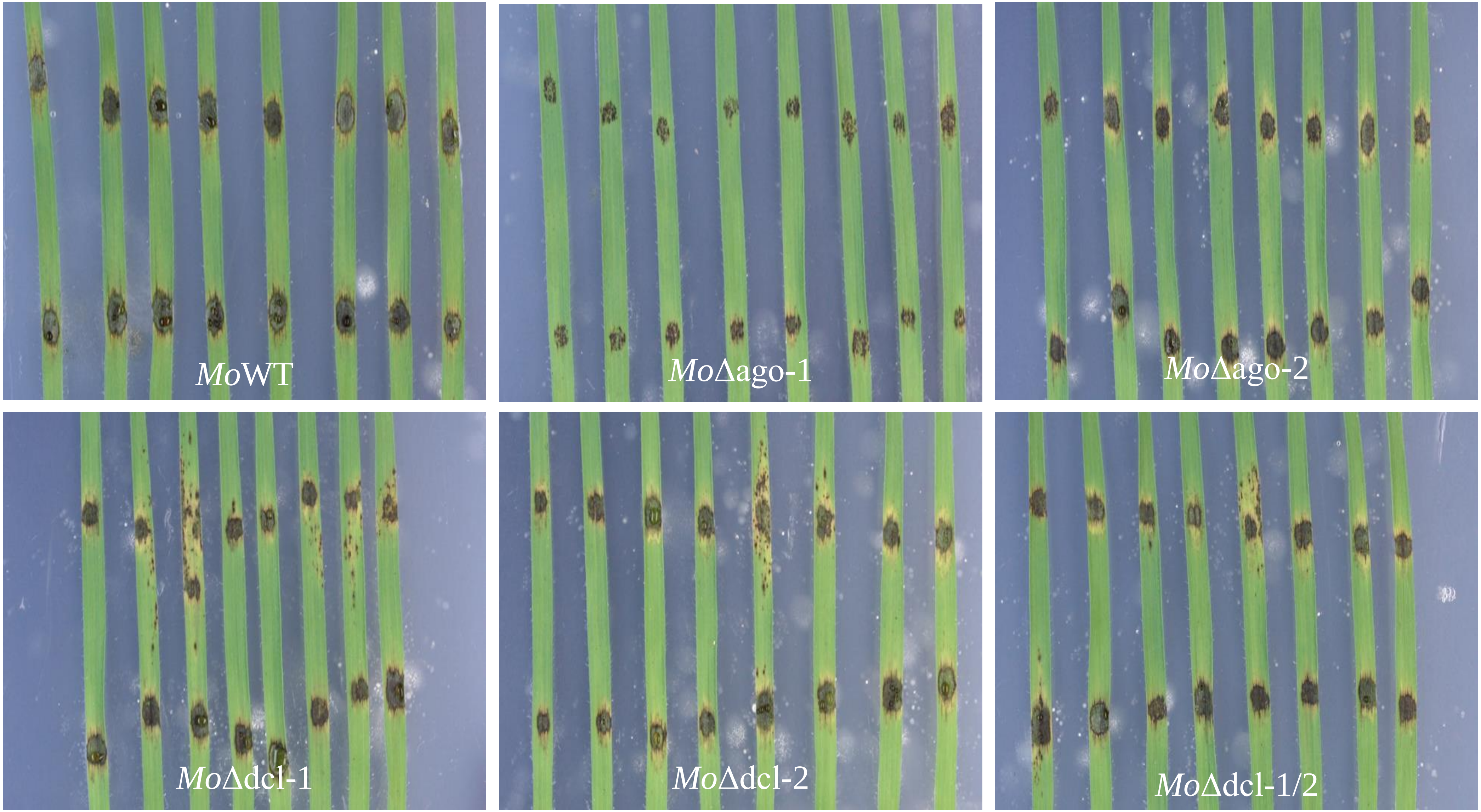

Figure S4 continuation

Detached leaves spray

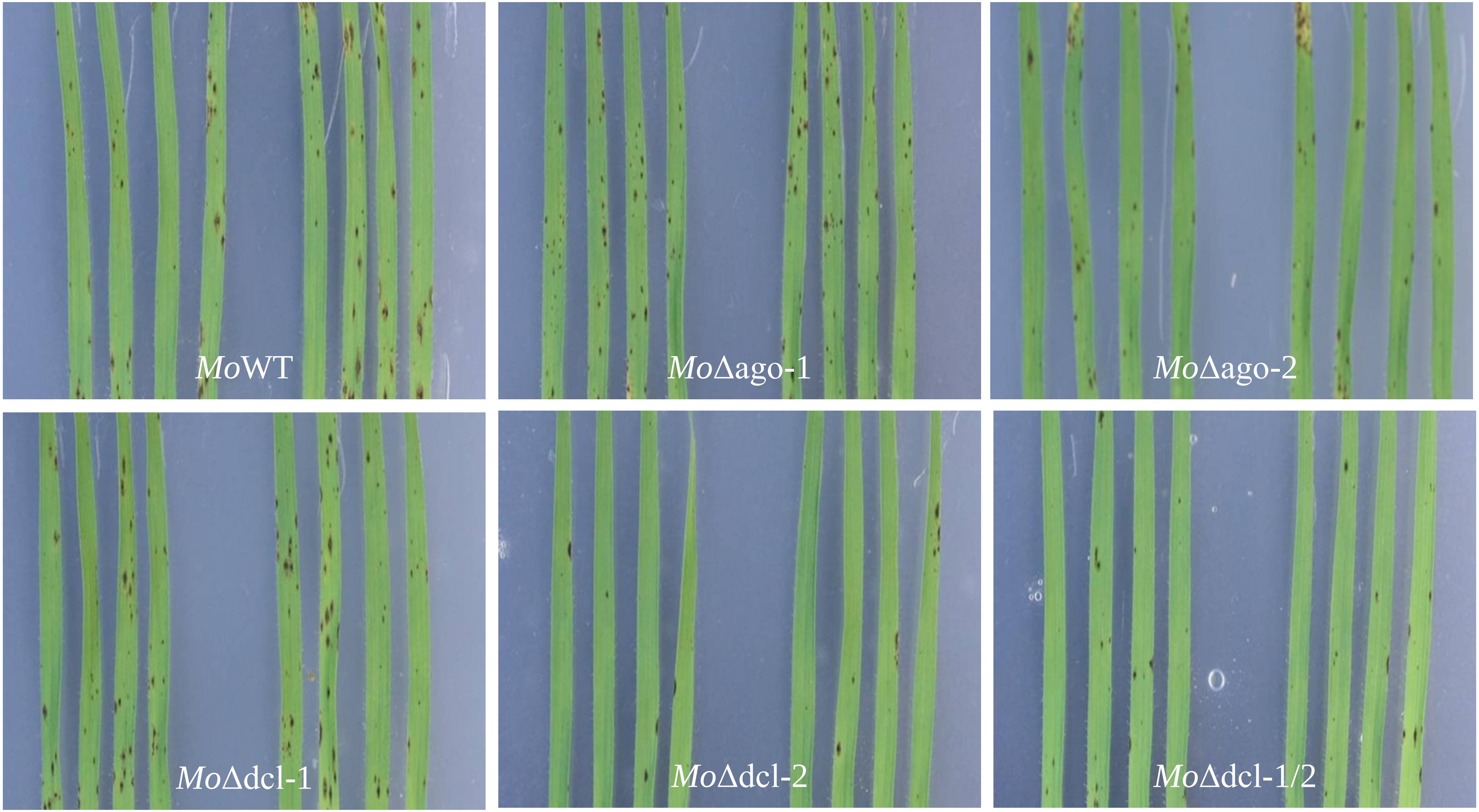

Figure S4 continuation

Root infection assay

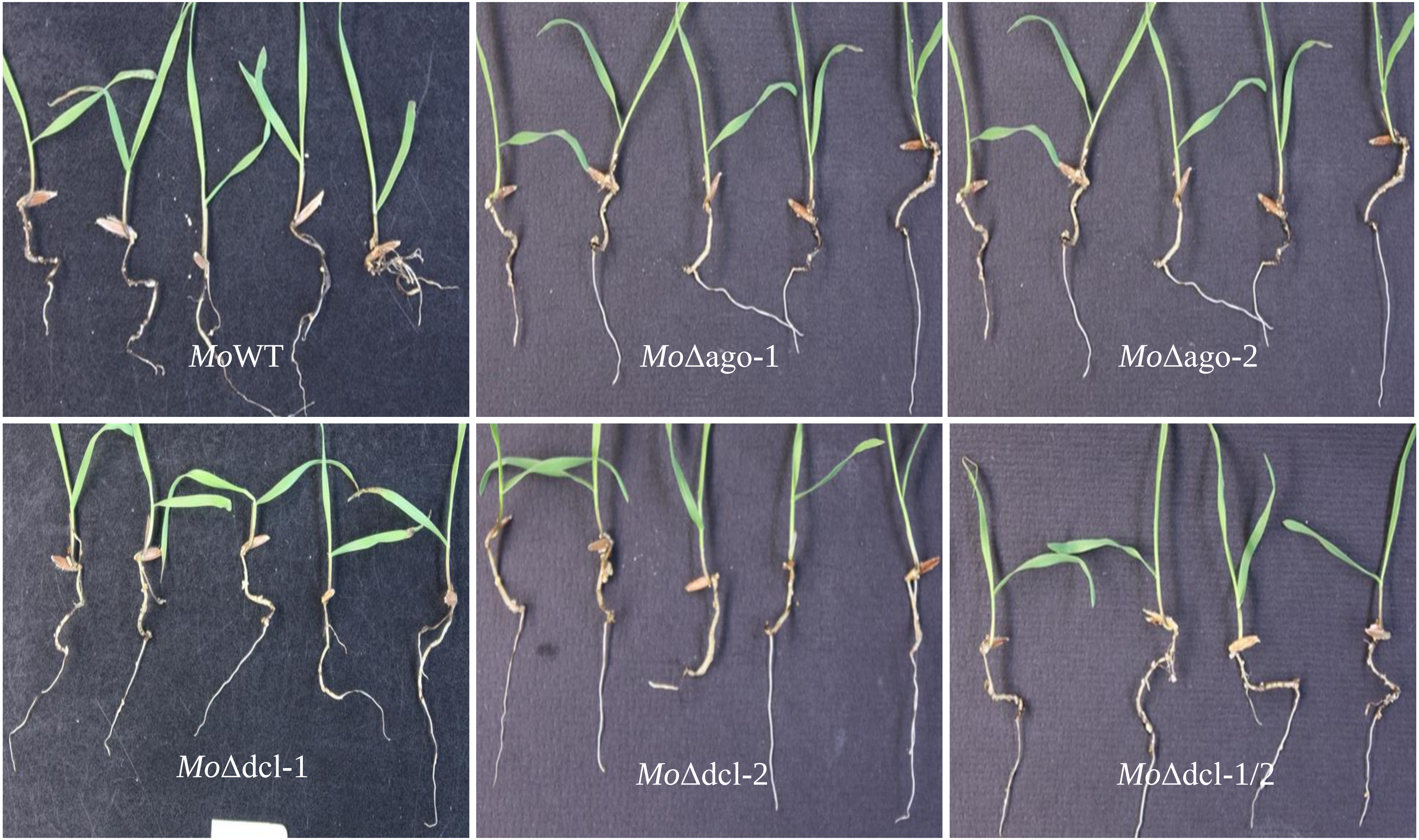

Figure S4 continuation

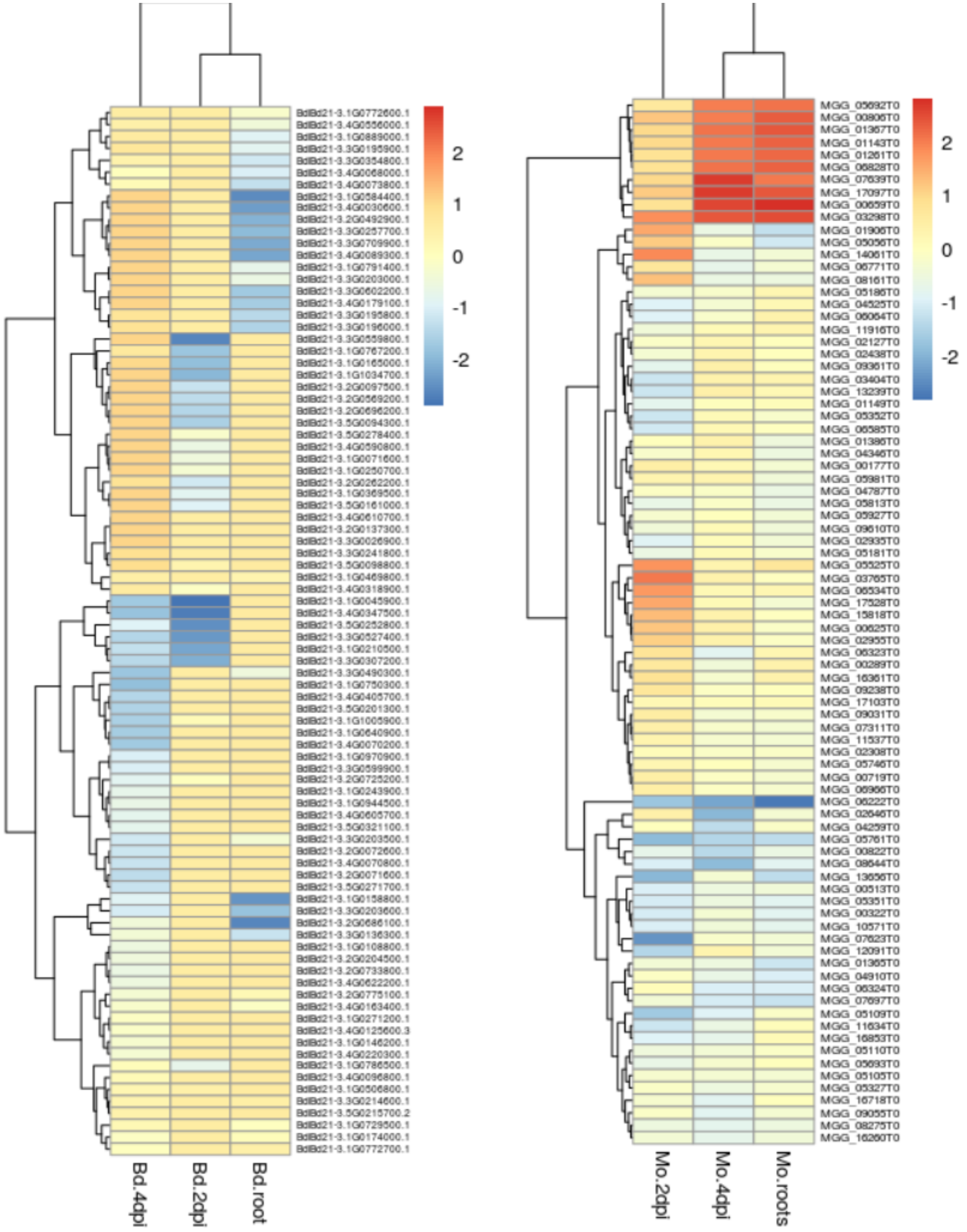

Figure S5. Heatmap for Mo and Bd DEG calling with DESeq2. Heatmap of expression levels (logFC) of the top Bd (A) and Mo (B) mRNAs in all 3 setups (leaf 2 DPI, leaf 4 DPI and root). Color gradient from red to blue indicative of log2FC of corresponding transcript as shown in the legend.

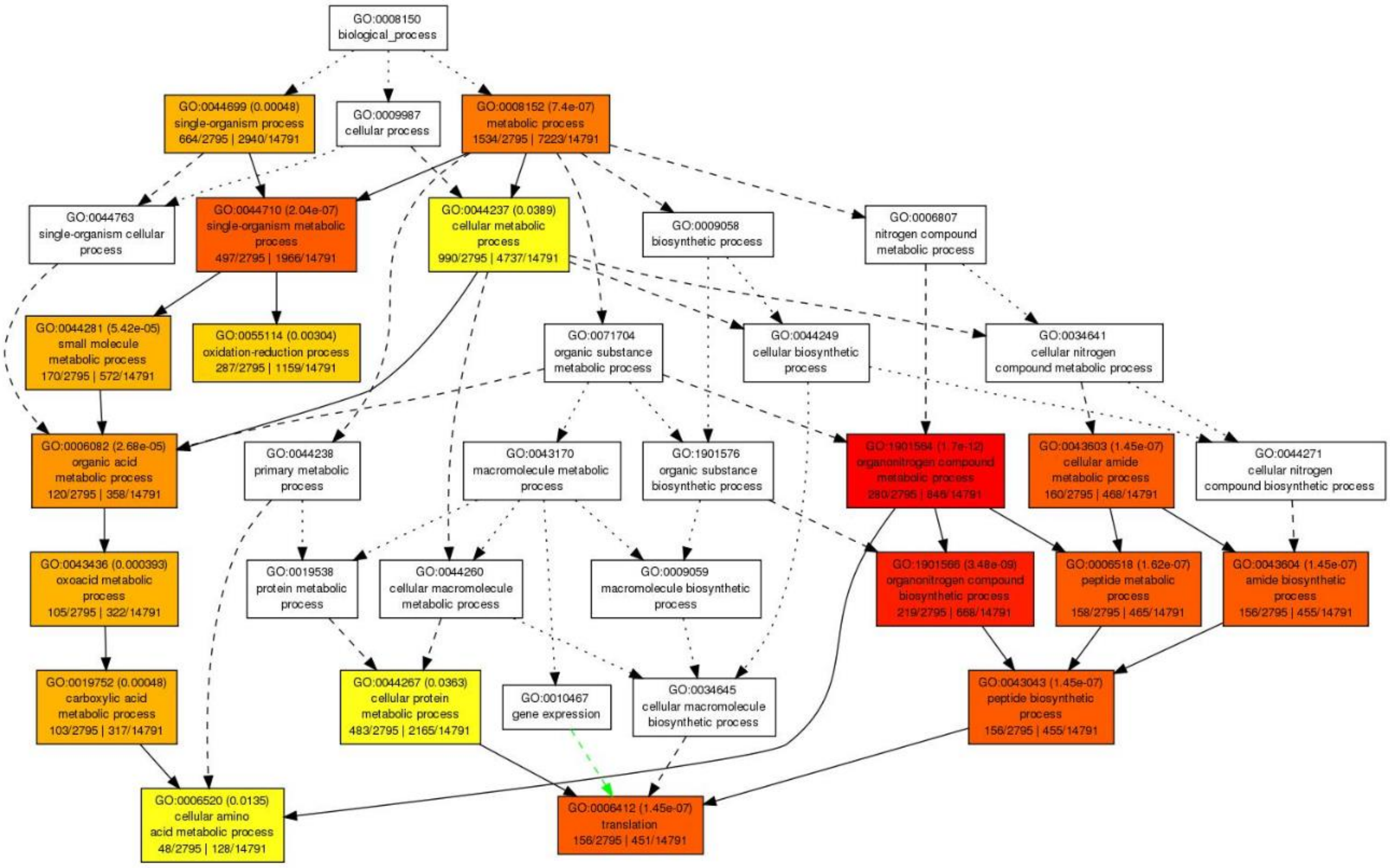

Figure S6. Results of gene ontology enrichment (GOE) analysis for significantly DE Bd genes in the 4 DPI leaf setup. GOE analysis done with AgriGO v2.

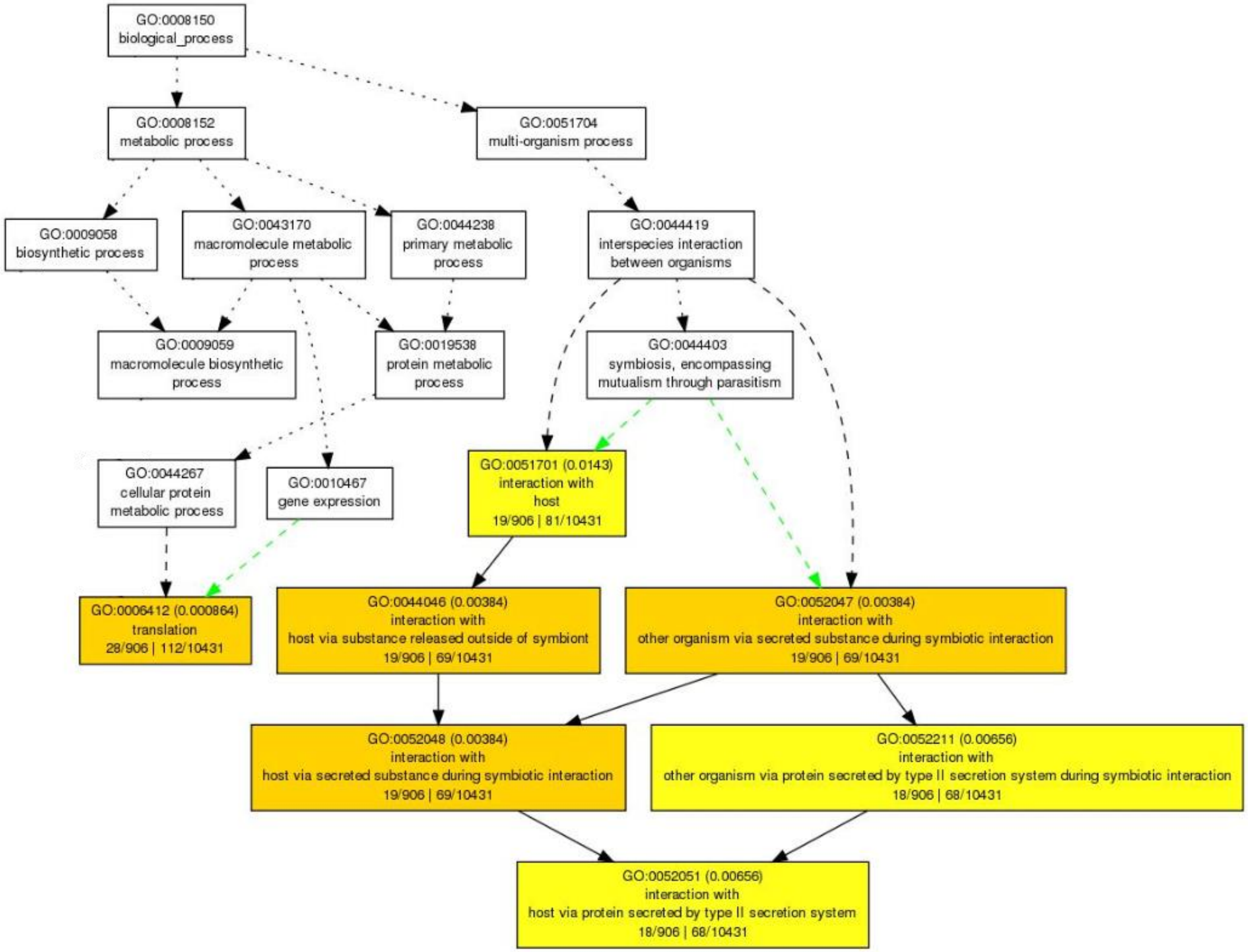

Figure S7. Results of gene ontology enrichment (GOE) analysis for significantly DE Mo genes in the root setup. GOE analysis done with AgriGO v2.

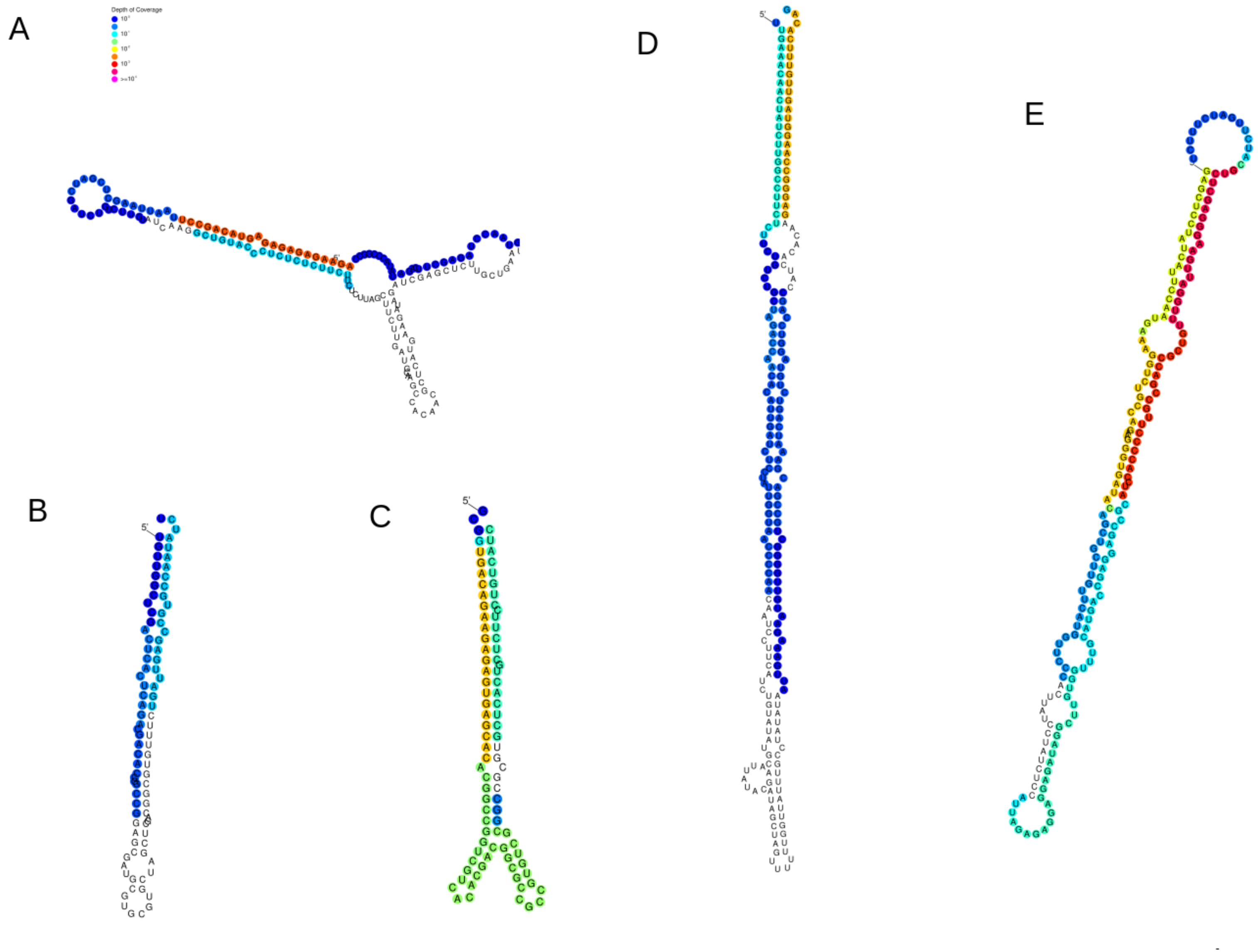

Figure S8. Visual representation of the identified upregulated Bd clusters (miRNA precursors) structures: (A) cluster_7470, (B) cluster_3162, (C) cluster_7744, (D) cluster_2384, (E) cluster_3312.
