# Supplementary material for: Comparative Analysis of Transcriptome and sRNAs Expression Patterns in the Brachypodium distachyon—Magnaporthe oryzae Pathosystems
Source: Int J Mol Sci. 2021 Jan 11;22(2):650. doi: 10.3390/ijms22020650 (PMC7826919; doi:10.3390/ijms22020650)
Supplement: Supplementary file 1 [file ijms-22-00650-s001.zip › Zanini_et_al_2020_Supplementary_tables.pdf]

Supplementary Table 1. Domain structures and coordinates (with corresponding pvalues in brackets) of MoAGOs and MoDCLs as detected by SMART+PFAM search.

Protein	N domain	DUF1785	PAZ	L2	PIWI
**MoAGO1**	150-287<br>(8.2e-16)	296-354<br>(5.54e-10)	392-515<br>(1.5e-15)	524-570<br>(4.3e-14)	672-970<br>(9.69e-100)
**MoAGO2**	56-200<br>(3.6e-19)	211-283<br>(4.36e-17)	352-439<br>(1.9e-9)	448-497<br>(9.7e-10)	606-969<br>(6.62e-84)
**MoAGO3**	172-314<br>(2.3e-14)	328-383<br>(5.09e-9)	387-534<br>(4.1e-8)	543-592<br>(8.8e-15)	702-1020<br>(1.4e-96)

Protein	DEXDc	HELICc	Dicer_dimer	RIBOc	RIBOc
**MoDCL1**	104-317<br>(6.29e-19)	436-565<br>(4.3e-19)	639-726<br>(8.4e-20)	1083-1190<br>(3.4e-19)	1258-1429<br>(4.73e-20)
**MoDCL2**	61-262<br>(3.17e-15)	415-541<br>(9.7e-18)	609-709<br>(4e-22)	1004-1142<br>(2.3e-14)	1183-1381<br>(3.99e-18)

Supplementary Table 2. MoAGOs and MoDCLs protein localization prediction results by PSI. Scores range from 0 to 1 and represent the likelihood of detecting the protein in the identified sublocalization, with corresponding pvalues.

Protein	Name	Gene	Sublocalization	Score	pvalue
**XP_003714515.1**	MoDCL1	MGG_01541	Nuclear	0.799	0
**XP_003715365.1**	MoDCL2	MGG_12357	Cytosol	0.565	0
**XP_003716704.1**	MoAGO1	MGG_14873	Nuclear	0.556	1.7435e-227
			Cytosol	0.329	1.5233e-100
**XP_003717504.1**	MoAGO2	MGG_13617	Cytosol	0.516	0
			Plastid	0.236	2.5706e-294
**XP_003714217.1**	MoAGO3	MGG_01294	Nuclear	0.747	0

Supplementary Table 3. Prediction of protein interactome for MoDCLs and MoAGOs with STRING. The score is the combination of experimental and co-expression scores from STRING prediction.

Interacting protein	Protein description	DCL1 score	DCL2 score
**MGG_01294**	Argonaute 3	0.664	0.517
**MGG_04429**	ATP-dependent DNA helicase MPH1	0.515	0.515
**MGG_11029**	Argonaute 1	0.664	0.512
**MGG_13617**	Argonaute 2	0.664	0.512
**MGG_02598**	30S ribosomal protein S16	0.463	0.463
**MGG_09464**	Uncharacterized protein	0.457	0.457
**MGG_06727**	WD domain-containing protein	0.45	0.45
**MGG_06468**	Uncharacterized protein	0.439	0.439

**MGG_09301**	Uncharacterized protein	0.416	0.416
**MGG_04741**	Small nuclear ribonucleoprotein Sm D1	0.503	
**MGG_09464**	Uncharacterized protein	0.457	
**MGG_07168**	Uncharacterized protein		0.551

Interacting protein	Protein description	AGO1 score	AGO2 score	AGO3 score
**DCL1**	Dicer-like protein 1	0.664	0.664	0.664
**DCL2**	Dicer-like protein 2	0.512	0.512	0.517
**MGG_13500**	U5 small nuclear ribonucleoprotein component	0.685	0.506	0.506
**MGG_06309**	Cell cycle control protein cwf14	0.685	0.506	0.506
**MGG_05172**	Uncharacterized protein	0.688		
**MGG_03158**	Pumilio-family RNA binding repeat protein	0.656		
**MGG_02762**	ATP-dependent RNA helicase DED1	0.64		
**MGG_13651**	High-affinity glucose transporter	0.614		
**MGG_06873**	Uncharacterized protein	0.611		
**MGG_13734**	Uncharacterized protein	0.611		
**MGG_15299**	Uncharacterized protein	0.611		

Supplementary Table 4. Expression of RNAi components during infection. BaseMean, LogFC and corresponding padj values of each RNAi component were retrieved from DESeq2 analyses of infected datasets: leaf 2 DPI, leaf 4 DPI and root. #N/A = not detected

	Leaf 2 DPI			Leaf 4 DPI			Root		
**Gene**	**Base Mean**	**log_2FC**	**padj**	**Base Mean**	**log_2FC**	**padj**	**Base Mean**	**log_2FC**	**padj**
**BdAGO1**	#N/A	#N/A	#N/A	#N/A	#N/A	#N/A	0.66	-1.38	NA
**BdAGO2**	8.18	-0.65	0.9999	6.45	-0.35	0.8330	26.00	0.29	0.9998
**BdAGO3**	853.25	-0.10	0.9999	1171.88	-0.38	0.0392	1151.24	0.11	0.9998
**BdAGO4**	#N/A	#N/A	#N/A	#N/A	#N/A	#N/A	#N/A	#N/A	#N/A
**BdAGO5**	#N/A	#N/A	#N/A	#N/A	#N/A	#N/A	0.19	1.04	NA
**BdAGO6**	0.82	1.02	NA	1.34	-0.41	0.9154	66.62	-0.02	0.9998
**BdAGO7**	#N/A	#N/A	#N/A	#N/A	#N/A	#N/A	#N/A	#N/A	#N/A
**BdAGO8**	0.21	0.98	NA	0.28	-1.27	NA	13.24	-0.14	0.9998
**BdAGO9**	48.27	0.22	0.9999	19.68	0.22	0.8161	241.50	-0.12	0.9998
**BdAGO10**	3.51	0.60	0.9999	2.60	1.28	0.5207	3.34	0.35	NA
**BdAGO11**	#N/A	#N/A	#N/A	#N/A	#N/A	#N/A	#N/A	#N/A	#N/A
**BdAGO12**	3.93	1.27	0.9999	7.49	1.19	0.2664	28.58	0.05	0.9998
**BdAGO13**	1.04	0.72	NA	0.92	-0.35	NA	8.21	0.10	0.9998
**BdAGO14**	15.03	0.53	0.9999	14.31	0.33	0.7539	91.33	0.03	0.9998
**BdAGO15**	#N/A	#N/A	#N/A	#N/A	#N/A	#N/A	21.55	-0.67	0.9998
**BdAGO16**	2.54	0.88	0.9999	2.05	1.51	0.5141	1.15	0.51	NA
**BdDCL1**	590.26	0.17	0.9999	962.05	0.50	0.0092	369.90	0.02	0.9998
**BdDCL2a**	241.16	0.18	0.9999	306.60	0.58	0.0042	301.78	-0.01	0.9998
**BdDCL2b**	1.01	0.39	NA	2.21	0.01	0.9988	41.90	0.05	0.9998
**BdDCL3a**	#N/A	#N/A	#N/A	#N/A	#N/A	#N/A	#N/A	#N/A	#N/A

**BdDCL3b**	123.58	-0.11	0.9999	136.99	-0.69	0.0179	193.75	0.08	0.9998
**BdDCL4**	#N/A	#N/A	#N/A	#N/A	#N/A	#N/A	#N/A	#N/A	#N/A
**MoAGO1**	1.43	-0.60	0.8495	7.83	-1.91	0.1300	0.84	0.96	0.8336
**MoAGO2**	1.42	-0.60	0.8560	8.92	-1.13	0.3495	2.67	1.93	0.4623
**MoAGO3**	74.63	-0.09	0.9116	425.73	0.69	0.0035	65.21	0.99	0.1353
**MoDCL1**	0.67	0.49	NA	3.70	-1.81	0.3272	0.40	2.03	NA
**MoDCL2**	13.13	0.01	0.9954	69.69	0.54	0.2343	7.53	0.49	0.8699

Supplementary Table 5. Selection of significantly enriched Bd GO terms from 2 DPI, 4 DPI and root Bd DEG datasets. GO terms and GOE analysis was done with AgriGO v2 against the pre-compiled Bd reference, after transforming Bd21-3 IDs to Bd21 ones. Abbreviations: O= ontology, P= biological process, F = molecular function, C = cellular component, BG = background, FDR = false discovery rate.

Setup	GO term	O	Description	Input list	BG/ Ref	p-value	FDR
**4 DPI**	GO:1901564	P	organonitrogen compound metabolic process	280	846	6.3E-16	1.7E-12
**4 DPI**	GO:1901566	P	organonitrogen compound biosynthetic process	219	668	2.6E-12	3.5E-09
**4 DPI**	GO:0006412	P	translation	156	451	1.9E-10	0.00000015
**4 DPI**	GO:0043603	P	cellular amide metabolic process	160	468	2.2E-10	0.00000015
**4 DPI**	GO:0043043	P	peptide biosynthetic process	156	455	3.2E-10	0.00000015
**4 DPI**	GO:0006518	P	peptide metabolic process	158	465	4.2E-10	0.00000016
**4 DPI**	GO:0008152	P	metabolic process	1534	7223	2.5E-09	0.00000074
**4 DPI**	GO:0006082	P	organic acid metabolic process	120	358	0.000000099	0.000027
**4 DPI**	GO:0044281	P	small molecule metabolic process	170	572	0.00000022	0.000054
**4 DPI**	GO:0043436	P	oxoacid metabolic process	105	322	0.0000017	0.00039
**4 DPI**	GO:0044699	P	single-organism process	664	2940	0.0000024	0.00048
**4 DPI**	GO:0055114	P	oxidation-reduction process	287	1159	0.000017	0.003
**4 DPI**	GO:0044267	P	cellular protein metabolic process	483	2165	0.00023	0.036
**4 DPI**	GO:0003735	F	structural constituent of ribosome	127	312	2.3E-12	1.7E-09
**4 DPI**	GO:0005198	F	structural molecule activity	133	330	1.2E-12	1.7E-09
**4 DPI**	GO:0003824	F	catalytic activity	1408	6501	2.4E-10	0.00000012
**4 DPI**	GO:0016491	F	oxidoreductase activity	318	1274	0.0000032	0.0012
**4 DPI**	GO:0035639	F	purine ribonucleoside triphosphate binding	376	1608	0.00006	0.015
**4 DPI**	GO:0005737	C	cytoplasm	239	793	2.3E-10	0.00000003
**4 DPI**	GO:0043228	C	non-membrane-bounded organelle	156	547	0.0000005	0.00038
**2 DPI**	GO:0055114	P	oxidation-reduction process	30	1159	5.70E-08	2.10E-05
**2 DPI**	GO:0044710	P	single-organism metabolic process	40	1966	1.40E-07	2.50E-05

**2 DPI**	GO:0044699	P	single-organism process	46	2940	1.90E-05	0.0023
**2 DPI**	GO:0005506	F	iron ion binding	20	313	7.20E-12	1.50E-09
**2 DPI**	GO:0016705	F	oxidoreductase activity, acting on paired donors	19	301	3.10E-11	3.10E-09
**2 DPI**	GO:0020037	F	heme binding	21	438	3.30E-10	2.20E-08
**2 DPI**	GO:0003824	F	catalytic activity	91	6501	5.70E-10	2.30E-08
**2 DPI**	GO:0046906	F	tetrapyrrole binding	21	452	5.70E-10	2.30E-08
**2 DPI**	GO:0016491	F	oxidoreductase activity	30	1274	4.30E-07	1.40E-05
**2 DPI**	GO:0043167	F	ion binding	31	1701	4.70E-05	0.0014
**2 DPI**	GO:0043169	F	cation binding	29	1601	9.90E-05	0.002
**Root**	GO:0006979	P	response to oxidative stress	8	169	4.80E-06	0.00087
**Root**	GO:0044710	P	single-organism metabolic process	25	1966	2.70E-05	0.001
**Root**	GO:0044264	P	cellular polysaccharide metabolic process	5	61	2.70E-05	0.001
**Root**	GO:0006950	P	response to stress	11	473	5.30E-05	0.0016
**Root**	GO:0005976	P	polysaccharide metabolic process	5	73	6.20E-05	0.0016
**Root**	GO:0055114	P	oxidation-reduction process	17	1159	0.00014	0.0032
**Root**	GO:0044262	P	cellular carbohydrate metabolic process	5	108	0.00035	0.0071
**Root**	GO:0016684	F	oxidoreductase activity, acting on peroxide as acceptor	8	174	5.90E-06	0.00029
**Root**	GO:0020037	F	heme binding	12	438	4.70E-06	0.00029
**Root**	GO:0003824	F	catalytic activity	56	6501	2.40E-06	0.00029
**Root**	GO:0046906	F	tetrapyrrole binding	12	452	6.50E-06	0.00029
**Root**	GO:0004601	F	peroxidase activity	8	171	5.20E-06	0.00029
**Root**	GO:0016830	F	carbon-carbon lyase activity	5	72	5.80E-05	0.0019
**Root**	GO:0016491	F	oxidoreductase activity	18	1274	0.00014	0.0039
**Root**	GO:0016829	F	lyase activity	6	174	0.00041	0.01
**Root**	GO:0016798	F	hydrolase activity, acting on glycosyl bonds	8	371	0.00097	0.022

Supplementary Table 6. Selection of significantly enriched Mo GO terms from 2 DPI, 4 DPI and root Mo DEG datasets. GO terms and GOE analysis was done with AgriGO v2 against the pre-compiled Mo reference. Abbreviations: P= biological process, F = molecular function, C = cellular component, BG = background, FDR = false discovery rate.

Setup	GO term	O	Description	Input list	BG/ Ref	p-value	FDR
**Root**	GO:0044408	P	growth or development of symbiont on or near host	198	1478	1.8E-09	0.00000065
**Root**	GO:0006412	P	translation	28	112	0.0000047	0.00086
**Root**	GO:0052047	P	interaction with other organism via secreted substance during symbiotic interaction	19	69	0.000052	0.0038
**Root**	GO:0044046	P	interaction with host via substance released outside of symbiont	19	69	0.000052	0.0038
**Root**	GO:0052048	P	interaction with host via secreted substance during symbiotic interaction	19	69	0.000052	0.0038
**Root**	GO:0052051	P	interaction with host via protein secreted by type II secretion system	18	68	0.00012	0.0066
**Root**	GO:0052211	P	interaction with other organism via protein secreted by type II secretion system	18	68	0.00012	0.0066
**Root**	GO:0051701	P	interaction with host	19	81	0.00031	0.014
**Root**	GO:0003735	F	structural constituent of ribosome	17	65	0.00022	0.024
**Root**	GO:0005198	F	structural molecule activity	17	71	0.00052	0.028
**2 DPI**	GO:0032502	P	developmental process	263	1048	8.50E-07	0.00066
**2 DPI**	GO:0048856	P	anatomical structure development	245	989	4.80E-06	0.0019
**2 DPI**	GO:0032501	P	multicellular organismal process	233	951	1.60E-05	0.0031
**2 DPI**	GO:0007275	P	multicellular organismal development	233	950	1.50E-05	0.0031
**2 DPI**	GO:0043581	P	mycelium development	230	941	2.10E-05	0.0033
**2 DPI**	GO:0034641	P	cellular nitrogen compound metabolic process	38	90	2.80E-05	0.0037
**4 DPI**	GO:0044408	P	growth or development of symbiont on or near host	521	1478	1.80E-08	1.50E-05

Supplementary Table 7. Overview of total sRNA and mRNA reads in the *Brachypodium distachyon* – *Magnaporthe oryzae* interaction. Total (tot.) reads sRNA: total number of raw sRNA reads per sample. Total sRNA reads Bd/ Total sRNA reads Mo: total number of sRNA reads aligned without mismatches to organism of origin genome that have at least 2 mismatches to the interacting organism genome. Total reads mRNA: total number of raw mRNA reads per sample.

Sample	Tot. reads sRNA	Tot. sRNA reads *Bd*	Tot. sRNA reads *Mo*	Tot. mRNA reads (millions)
**2 DPI leaves con**	28 435 036	18 691 859		59.5
**2 DPI leaves *Mo***	35 544 788	25 844 498	62 709	59.3
**4 DPI leaves con**	21 846 438	15 214 694		105
**4 DPI leaves *Mo***	26 376 527	15 623 561	827 678	82.8
**Roots con**	33 722 475	23 139 570		47.8
**Roots *Mo***	37 697 368	26 378 677	96 506	44.9
**Axenic culture *Mo***	26 407 359		13 721 649	65
